# Quantitative analysis of chaperone network throughput in budding yeast

**DOI:** 10.1002/pmic.201200412

**Published:** 2013-03-15

**Authors:** Philip Brownridge, Craig Lawless, Aishwarya B Payapilly, Karin Lanthaler, Stephen W Holman, Victoria M Harman, Christopher M Grant, Robert J Beynon, Simon J Hubbard

**Affiliations:** 1 Protein Function Group Institute of Integrative Biology, University of LiverpoolLiverpool, UK; 2 Faculty of Life Sciences, University of ManchesterManchester, UK; 3 Michael Barber Centre for Mass Spectrometry School of Chemistry, University of ManchesterManchester, UK

**Keywords:** Chaperones, Protein folding, Protein interactions, QconCAT, Quantitative proteomics, Systems biology

## Abstract

The network of molecular chaperones mediates the folding and translocation of the many proteins encoded in the genome of eukaryotic organisms, as well as a response to stress. It has been particularly well characterised in the budding yeast, *Saccharomyces cerevisiae*, where 63 known chaperones have been annotated and recent affinity purification and MS/MS experiments have helped characterise the attendant network of chaperone targets to a high degree. In this study, we apply our QconCAT methodology to directly quantify the set of yeast chaperones in absolute terms (copies per cell) via SRM MS. Firstly, we compare these to existing quantitative estimates of these yeast proteins, highlighting differences between approaches. Secondly, we cast the results into the context of the chaperone target network and show a distinct relationship between abundance of individual chaperones and their targets. This allows us to characterise the ‘throughput’ of protein molecules passing through individual chaperones and their groups on a proteome-wide scale in an unstressed model eukaryote for the first time. The results demonstrate specialisations of the chaperone classes, which display different overall workloads, efficiencies and preference for the sub-cellular localisation of their targets. The novel integration of the interactome data with quantification supports re-estimates of the level of protein throughout going through molecular chaperones. Additionally, although chaperones target fewer than 40% of annotated proteins we show that they mediate the folding of the majority of protein molecules (∼62% of the total protein flux in the cell), highlighting their importance.

## 1 Introduction

One of the goals of quantitative proteomics is to characterise the level of protein in a cellular system in order to understand how the gene products are organised and regulated. Such understanding underpins any comprehensive systems model of an organism and its biological functions, where genome, transcriptome, proteome and metabolome interact with each other to maintain homeostasis or react to stress and perturbation. This in turn builds on the classical molecular biology ‘dogma’ coined by Crick, where ‘DNA makes RNA makes protein’, which now includes a more complex model involving multiple isoforms and interactions. For example, recent studies have characterised the entire yeast transcriptome 1, interactome 2,3, measured translational control rates 4, protein locations 5 and half-lives 6. Quantitative proteomics has targeted *Saccharomyces cerevisiae* (‘yeast’) as a model organism and several proteome-wide datasets exist 7–9. Similarly, in mammalian cells, great strides have been made in the integration of transcription, translation and turnover of both RNA and protein to build genome-scale models 10. This epitomises the challenges facing systems biology where integration of such information is needed to understand the full complexities of biological control and regulation of function.

Although such studies now build protein abundance and even half-life into the model, in order for a given protein to function it also needs to be folded, active, and delivered to its site of action. The proteins responsible for this are the chaperones, of which 63 are known in yeast 11. They operate as individual proteins or assembled into molecular machines, to recognise their targets, promote the correct folding and help deliver them to their sub-cellular destination 12. They play a vital role in preventing protein aggregation by recognising the nascent peptide chain to ensure proper folding in a biologically meaningful timescale. Chaperones are also involved in other linked areas, including ribosomal RNA processing, translocation across membranes and cellular response to stress 13. There are 63 yeast chaperones including the so-called heat-shock proteins: Hsp100, Hsp90, Hsp70, Hsp60 and the smaller HSPs that are ubiquitous in eukaryotic cells, and much is known about the mechanistic details of individual chaperones at the molecular level. However, a comprehensive understanding of the cellular roles played by chaperones is only just emerging. Recent pioneering work using affinity purification coupled to MS has defined a comprehensive *qualitative* dataset describing chaperone–chaperone and chaperone-target interactions for all 63 yeast chaperones 11 but we know little regarding the changes in these networks during stress conditions, or when recombinant protein expression perturbs the system.

Our understanding of chaperone networks and their properties is emerging 11,14,15. Frydman and colleagues demonstrated that two distinct and broad chaperones classes carry out different generic fundamental roles, delivered via common regulatory properties 14. More recently, an analysis of chaperone interactome data addresses the scope of individual chaperone systems by clustering the chaperone-target network into modules that show some conserved properties, including evolutionary rates 16. These modules are quite different from the expected chaperone classes described above and strongly support the hypothesis that chaperones act in distinct communities, targeted at selected protein groups.

Here we extend the previous studies, adding further quantitative data to this network via QconCAT and other extant quantitative datasets available in the public domain, including target protein degradation rates. We show that there is a correlation between chaperone abundance and the workload each chaperone has in the yeast cell, represented by the number of known interactors, the abundance of their targets, and the estimated folding flux. We also consider the total flux through each chaperone (and chaperone group) and consider this in the context of annotated biological function. We discuss this in terms of sub-cellular localisation and previously reported throughput in chaperone pathways, as well as essentiality of protein targets. This represents a first look at the total folding flux placed upon the chaperone network derived from quantitative proteomics data and highlights the global role they play in regulating protein folding.

## 2 Materials and methods

### 2.1 Yeast and QconCAT sample preparation

The QconCAT proteins were produced as previously described 17 using cell lysis by sonication and purified by Ni-MAC nickel affinity column (Novagen, Merck Millipore).

*S. cerevisiae* (EUROSCARF accession number Y11335 BY4742; *Mat ALPHA*; *his3Δ1*; *leu2Δ0*; *lys2Δ0*; *ura3Δ0; YJL088w::kanMX4*) was grown in defined minimal C-limiting (F1) medium 18 using 10 g/L of glucose as the sole carbon source. The F1 medium was additionally supplemented with 0.5 mM arginine and 1 mM lysine to meet the added auxotrophic requirements of the strain. For biological replication, four cultures were grown in chemostat mode at a dilution rate of 0.1 h^−1^ and aliquots (15 mL) of the culture were centrifuged (4000 rpm; 4°C; 10 min). The supernatant was discarded, the pellet flash frozen in liquid nitrogen and stored at −80°C for subsequent protein extraction. Cell counts were performed using an automated cell counter (Cellometer AUTOM10 by Nexcelom. http://www.nexcelom.com). Proteins were extracted by re-suspending the biomass pellets in 250 μL of 50 mM ammonium bicarbonate (filter sterilised) containing 1 tablet of Roche complete-mini protease inhibitors (+EDTA) (Roche Diagnostics, West Sussex, UK) per 10 mL of ammonium bicarbonate. Acid-washed glass beads (200 μL) were added. The pellet was subjected to repeated bead-beating for 15 bursts of 30 s with a 1 min cool down in between each cycle. The biomass was centrifuged for 10 min at 13 000 rpm at 4°C; the supernatant was removed and stored in low bind tubes on ice. Fresh ammonium bicarbonate (250 μL) with protease inhibitors was added and the pellet re-suspended by vortexing. The bottom of the extraction vial was pierced with a hot needle, the vial placed on a fresh Eppendorf tube and quickly spun down (5 min at 4000 rpm at 4°C. The flow-through and the supernatant fraction were combined, the exact volume measured and the amount of protein determined by standard assay (Bio-Rad Laboratories, Hertfordshire, UK). Protein extracts were aliquoted and stored at −80°C prior to subsequent digestion.

The targets of QconCAT 1, 2 and 3 differ widely in abundance, whereas the peptides derived from each QconCAT are equimolar. Quantification therefore required multiple analytical runs at different loadings of QconCAT to constrain quantification to analyte:standard ratios between 10:1 and 1:10. To achieve this, the digestion strategy employed three separate digests for each bioreplicate, one codigest of yeast lysate with QconCAT and two unspiked digestions of yeast lysate. An amount of yeast lysate representing protein from 21.5 million cells was dispensed into low-protein-binding microcentrifuge tubes (Sarstedt, Leicester, UK) and made up to 150 μL by addition of 25 mM ammonium bicarbonate, and, in the case of the QconCAT codigests, 7 μL of QconCAT solution. The proteins were denatured using 10 μL of 1% w/v RapiGest™ (Waters, Manchester, UK) in 25 mM ammonium bicarbonate and followed by incubation at 80°C for 10 min. The sample was then reduced (addition of 10 μL of 60 mM DTT and incubation at 60°C for 10 min) and alkylated (addition of 10 μL of 180 mM iodoacetamide and incubation at room temperature for 30 min in the dark). To allow quantification of the QconCAT, 10 μL of 2.15 pmol/μL glu-fibrinopeptide (Waters) was added to each digest. Trypsin (Sigma, Poole, UK, proteomics grade) was reconstituted in 50 mM acetic acid to a concentration of 0.2 μg/μL and 10 μL added to the sample followed by incubation at 37°C. After 4.5 h an additional 10 μL of trypsin was added and the digestion left to proceed overnight. The digestion was terminated and RapiGest™ removed by acidification (3 μL of TFA and incubation at 37°C for 45 min) and centrifugation (15 000 × *g* for 15 min). To check for complete digestion and to quantify the QconCAT, each digest was analysed by LC-MS using a nanoAcquity UPLC™ system (Waters) coupled to a Synapt™ G2 mass spectrometer (Waters) in MS^E^ mode and searched against a sequence database (see Supporting Information). The QconCAT was quantified by integrating the peaks generated from XIC of *m/z* 785.8 (internal standard glu-fibrinopeptide) and *m/z* 788.8 (glu-fibrinopeptide from QconCAT digestion).

### 2.2 MS and data analysis

The database search results corresponding to the CopyCAT were converted into a spectral database using Skyline 19 and the four most intense fragment ions were selected as putative transitions. The [^12^C_6_] lys, arg analyte and [^13^C_6_] lys, arg standard equivalents of these four transitions were tested by application to both yeast only and yeast-QconCAT digest samples. SRM analysis was performed using a nanoAcquity UPLC™ system (Waters) coupled to a Xevo™ TQ triple quadrupole mass spectrometer (Waters) (see Supporting Information). The three transitions with the greatest S/N were selected for the final quantification analysis and optimised for collision energy and cone voltage. For final quantification, samples containing the protein equivalent of 200 000 cells with either 0.2, 2 or 20 fmol of QconCAT were analysed by the previously described SRM methodology. The dilution was prepared by serial dilution of the yeast-QconCAT codigest by the unspiked yeast digest – this reduced the complications of absorption of diluted standard peptides and ensured that the QconCAT peptides, regardless of concentration, were maintained in an abundant peptide environment. A set of decoy transitions were created according to 20 and run against the yeast digest under identical instrument parameters.

Label-free analysis was performed using a ‘Top3’ methodology 21 on a yeast digest not containing QconCAT. The label-free analysis was performed on two platforms, an ion-mobility coupled data independent (HDMS^E^) method on Synapt™ G2 (as previously described) and a data-dependent method on a Q-Exactive™ (Thermo Scientific, Hemel Hempstead, UK). HDMS^E^ acquisition was performed as 22. For the data-dependent method a portion (4 μL) of each yeast digest (100 000 cells/μL) was mixed with 1 μL of standard protein (50 fmol/μL rabbit muscle glycogen phosphorylase MassPREP™ Digestion Standard, Waters). The resulting spiked digests were analyzed by LC-MS using a Ultimate 3000 RSLC™ nano system (Thermo Scientific) coupled to a Q-Exactive™ mass spectrometer (Thermo Scientific) (see Supporting Information). The data were processed with Progenesis (version 4, Nonlinear Dynamics, Newcastle upon Tyne, UK). Samples were aligned according to retention time using a combination of manual and automatic alignment. Default peak picking parameters were applied and features with charges from 1^+^ to 4^+^ featuring three or more isotope peaks were retained. Database searching was performed using Mascot (Matrix Science, London, UK) (see Supporting Information). These identifications were imported into Progenesis and the resulting feature set was exported to ProgenPostProcessor 23 which can produce ‘Top N’ quantification values from Progenesis feature files. Based on the glycogen phosphorylase standard, Top 1, Top 2 and Top 3 based quantification was performed (depending on the number of peptides observed per protein, 75% of proteins were Top3) and the highest possible N used for final quantification. We refer to this dataset as Q-Exactive Hi3.

### 2.3 QconCAT Data processing and analysis

QconCAT quantification values were produced using the mProphet package 20. Decoy transitions were generated for each equivalent target transition using the provided mGen.pl Perl script using the SPIKE_IN workflow option. Waters *.raw* files were converted to the required *.mzXML* format using wolf-MRM (http://tools.proteomecenter.org/software/wolf-mrm/wolf-mrm.zip). The. *mzXML* files for both target and decoy data were merged using an in-house Perl script to align the acquired target and decoy transitions. Merged *.mzXML* files were passed through mMap.pl using *–mach TSQ* option with the transition list output produced using mGen.pl. The mMap.pl output was then submitted to mQuest.pl using an optimised parameter file (see Supporting Information). The output files from mQuest.pl were then submitted to mProphet.pl to produce the final light-heavy (target-standard) ratios and calculate-associated false discovery rates for all peptide quantifications. Final copies per cell (cpc) were then calculated from the analyte:standard ratio, known concentration of spike-in heavy standard and the number of cells loaded onto the column (obtained from a cell count performed on each sample). Peptide quantification values were then used only for those where at least three out of four biological replicates passed at 1% false discovery rate and all of which had an S/N >5.

## 3 Results and discussion

### 3.1 Absolute quantification of 63 chaperones

**Figure 1:**
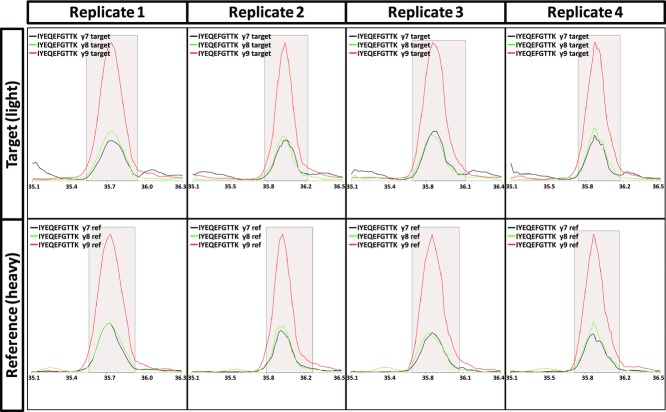
Extracted XICs from mProphet for three selected transitions for both light (yeast analyte) and heavy (QconCAT surrogate) peptide sequence IYEQEFGTTK. Grey boxes represent the peak group from which areas are calculated for quantification. All four bioreplicate XICs are shown displaying excellent reproducibility. The parent protein, CCT5, was not observed in either of the epitope-tagging datasets available 8,9.

Using our QconCAT methodology 24, as part of a larger scale project to quantify in absolute terms the entire cellular proteome of *S. cerevisiae* 25, we designed three recombinant proteins constituting concatamers of surrogate peptides to enable the quantification of the 63 annotated chaperones in the yeast genome. These recombinant proteins were expressed in media supplemented with stable isotope labelled amino acids to generate ‘heavy’ QconCAT proteins that were then spiked into yeast protein samples to enable absolute quantification in copies per cell, across four biological replicates. Two peptides were nominated to quantify each protein. For each peptide, cpc values were averaged across replicates where available. Protein abundances were then calculated from the two mean values, taken as the maximum of the two peptides. Such protein quantifications are termed Type A, where acceptable data are available for both analyte and surrogate peptides. In other instances, the analyte quantotypic peptide was not observed although the QconCAT peptide was (Type B), and in a few, neither peptide extracted ion chromatograms (XICs) were observed above the minimum S/N (Type C). A complete list of the chaperone proteins with cpc is in Table 1 and details of peptides selected and individual peptide-level cpc values are in Supporting Information Table S1. Using our standard QconCAT method we obtained absolute quantitative values in cpc for 51 of the 63 chaperones. We updated our data analysis pipeline to incorporate the mProphet software 20, which supports semi-automated detection and quantification of chromatographic peaks and provides false discovery rate analysis from decoy transitions. Full details on the data processing to derive cpc are given in Section 2. Figure 1 shows representativeXICs for three transitions analysed for the peptide IYEQEFGTTK from Cct5 (further examples are provided in Supporting Information Fig 1A and B). This is a representative Type A quantification where good repeatability is apparent across four bioreplicates. We were able to quantify chaperones across a broad dynamic range from 250 to 440 000 cpc. There is good coverage of all the chaperone classes (including the CCT proteins that have proven refractive to epitope-tagging strategies) and we have succeeded in quantifying some proteins that other label-mediated strategies have missed (e.g. HSP70 protein Ssa3). In part, this is due to our targeted approach that selects unique peptides wherever possible, avoiding peptides shared between several closely related proteins. We are also able to offer an upper limit on three SMALL class chaperones that no other method has yet reported, according to PaxDB 26.

**Table 1:** Yeast chaperones and absolute abundance expressed in copies per cell (cpc). UniProt, SGD and systematic ORF names are provided along with QconCAT-based quantification. The quantification type refers to the status of the endogenous peptide and surrogate peptide XIC via mProphet analysis, classifying cases where the endogenous peptide is not observed above the S/N at any of the three spike-in concentrations as Type B, and cases where neither peptide is observed as Type C

Chaperone class	SGD gene name	Systematic name	UniProt	QconCAT (CPC ± SEM)a)	Type
SMALL	Hsp12	YFL014W	P22943	439 000 ± 35500	A
SMALL	Hsp26	YBR072W	P15992	235 000 ± 33 500	A
SMALL	Hsp42	YDR171W	Q12329	9150 ± 450	A
SMALL	Hsp31	YDR533C	Q04432	6450 ± 550	A
PFD	Gim4	YEL003W	P40005	9650 ± 300	A
PFD	Pac10	YGR078C	P48363	4750 ± 200	A
PFD	Gim3	YNL153C	P53900	4050 ± 350	A
PFD	Yke2	YLR200W	P52553	3050 ± 300	A
PFD	Pfd1	YJL179W	P46988	2950 ± 200	A
PFD	Gim5	YML094W	Q04493	2000 ± 100	A
HSP90	Hsc82	YMR186W	P15108	84 500 ± 2000	A
HSP90	Hsp82	YPL240C	P02829	23 000 ± 1000	A
HSP70	Ssa1	YAL005C	P10591	335 500 ± 25,500	A
HSP70	Ssb2	YNL209W	P40150	85 500 ± 3500	A
HSP70	Ssb1	YDL229W	P11484	68 500 ± 1500	A
HSP70	Ssz1	YHR064C	P38788	63 000 ± 3500	A
HSP70	Sse1	YPL106C	P32589	62 000 ± 2000	A
HSP70	Ssc1	YJR045C	P12398	58 000 ± 4000	A
HSP70	Ssa2	YLL024C	P10592	58 000 ± 2500	A
HSP70	Kar2	YJL034W	P16474	26 500 ± 1500	A
HSP70	Sse2	YBR169C	P32590	5700 ± 250	A
HSP70	Ssa4	YER103W	P22202	5700 ± 50	A
HSP70	Ssq1	YLR369W	Q05931	2300 ± 100	A
HSP70	Lhs1	YKL073W	P36016	2450 ± 300	A
HSP70	Ssa3	YBL075C	P09435	660 ± 55	A
HSP60	Hsp60	YLR259C	P19882	47 000 ± 2500	A
HSP40	Zuo1	YGR285C	P32527	31 500 ± 1500	A
HSP40	Sis1	YNL007C	P25294	15 000 ± 500	A
HSP40	Sec63	YOR254C	P14906	11 000 ± 500	A
HSP40	Caj1	YER048C	P39101	5000 ± 300	A
HSP40	Tim14	YLR008C	Q07914	4000 ± 100	A
HSP40	Erj5	YFR041C	P43613	3400 ± 100	A
HSP40	Mdj1	YFL016C	P35191	3200 ± 200	A
HSP40	Swa2	YDR320C	Q06677	1550 ± 50	A
HSP40	Scj1	YMR214W	P25303	1500 ± 100	A
HSP40	Djp1	YIR004W	P40564	1050 ± 100	A
HSP40	Jjj1	YNL227C	P53863	500 ± 80	A
HSP40	Jac1	YGL018C	P53193	470 ± 210	A
HSP40	Jem1	YJL073W	P40358	450 ± 40	A
HSP40	Xdj1	YLR090W	P39102	330 ± 20	A
HSP40	Mdj2	YNL328C	P42834	250 ± 50	A
CCT	Cct6	YDR188W	P39079	5300 ± 900	A
CCT	Cct4	YDL143W	P39078	20 000 ± 1000	A
CCT	Tcp1	YDR212W	P12612	11 500 ± 1000	A
CCT	Cct2	YIL142W	P39076	7050 ± 550	A
CCT	Cct5	YJR064W	P40413	6300 ± 150	A
CCT	Cct8	YJL008C	P47079	6000 ± 100	A
CCT	Cct7	YJL111W	P42943	2900 ± 100	A
AAA+	Hsp104	YLL026W	P31539	27 500 ± 2000	A
AAA+	Hsp78	YDR258C	P33416	10 500 ± 500	A
AAA+	Mcx1	YBR227C	P38323	1200 ± 250	A
HSP70	Ecm10	YEL030W	P39987	<600	B/B
HSP40	Cwc23	YGL128C	P52868	<600	B/B
HSP40	Jjj2	YJL162C	P46997	<6000	B/B
HSP40	Jjj3	YJR097W	P47138	<600	B/B
HSP40	Jid1	YPR061C	Q12350	<600	B/B
HSP40	Hlj1	YMR161W	P48353	<600	B/C
HSP40	Apj1	YNL077W	P53940	<6000	B/C
CCT	Cct3	YJL014W	P39077	<600	B/C
SMALL	Sno4	YMR322C	Q04902	<600	B/C
SMALL	Hsp33	YOR391C	Q08914	<600	B/C
SMALL	Hsp32	YPL280W	Q08992	<600	B/C
HSP40	Ydj1	YNL064C	P25491	–	C/C

a) An upper quantification limit can be placed for proteins with B-type peptides where no A-type peptides were available.

– indicates no quantification.

Errors across four biological replicates are expressed as SEM. All cpc and error values were rounded (to nearest 500 for cpc > 10 000, nearest 50 for cpc > 1000 and nearest 10 for lower cpc values).

**Figure 2:**
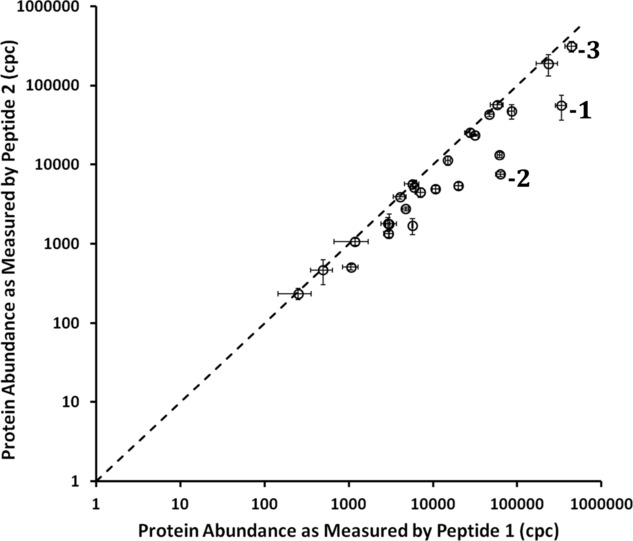
Sibling peptide *XY*-plot for all chaperone proteins quantified using two surrogate QconCAT peptides. The most abundant of the pair is along the *x*-axis. Large discrepancies between sibling peptide pairs can in part be explained by poor cleavage contexts. Peptide-1: The lower abundance sibling (Ssa1: AEETISWLDSNTTASK) for this protein contains a dibasic (KK) tryptic site at the N-terminus, of which a missed cleavage variant ([K]AEETISWLDSNTTASK) has been observed in subsequent mass spectrometric analysis. Additional signal loss may be attributed to the acidic residues surrounding the C-terminal cleavage site (Glu-Glu), which are known to increase the propensity of a missed cleave 43. Peptide-2: The peptide LNDAVEYVSGR from Hsp12 has an interspersed dibasic N-terminal tryptic site (KSK) that would affect proper cleavage 27,28. Peptide-3: The peptide LAAEDYIGSAVK from Ssz1 with lower abundance may be attributed to missed cleavage via an interspersed dibasic tryptic site at the N-terminal and glutamic acid immediately proceeding the C-terminal tryptic site.

The QconCAT approach benefits from inclusion of at least two peptides per protein that were observed *via* LC-MS for most of our targets, allowing comparison of sibling peptides from the same parent protein (Fig. 2). With few exceptions, there was good agreement between sibling peptides. The chaperone protein group was one of our first assemblies of designed QconCAT peptides and in retrospect, there were sub-optimal choices for some standard surrogate peptides. We have become increasingly aware of the importance of poor cleavage contexts in the standard of the endogenous yeast protein (Fig. 2) and we have observed the attendant missed cleavage peptides in subsequent MS analyses. These are typified by cases where dibasic sites or acidic amino acids lie close to or span the desired cleavage site (e.g. RR, KK, KR, RK, etc.), which are now deprecated in QconCAT design, and which should also inform any selection of proteotypic peptides 27,28. The peptide pairs in Fig. 2 are ordered to place the higher of the two abundance values on the *x*-axis. In all cases where one of a sibling pair is subtended by a missed cleavage site, its cpc quantification was lower than its partner because a significant fraction of the analyte peptide signal was lost to the miscleaved peptides that would manifest different retention times, masses and transitions. Some poor cleavage contexts are highlighted in Fig. 2, such as peptide 1 that is subtended by KK at the N-terminus in the parent protein.

### 3.2 Comparison with other protein quantification methods

**Figure 3:**
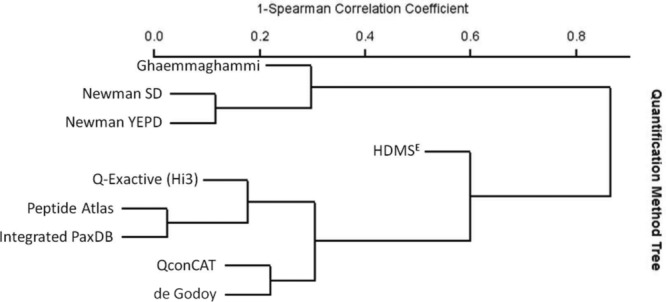
Dendogram of chaperone quantification values from different experimental methodologies. All but the QconCAT, Q-Exactive Hi3 and HDMS^E^ datasets were obtained from PaxDB 26. All protein abundance values are expressed as ppm for comparisons with PaxDB data, assuming 60 million protein cpc in total for the QconCAT, Q-Exactive Hi3 and HDMS^E^ datasets. Primary references for the independent datasets are: Ghaemmaghami 8, Newman SD and YEPD 9, and de Godoy 7.

As others and we have previously noted, there is a considerable discrepancy in the quantified abundance of proteins determined by alternative technologies. In Fig. 3 we compare the values obtained from our label-mediated QconCAT approach to other MS-based studies and to epitope-tagging approaches; Scatterplots for all pairwise comparisons were also generated (Supporting Information >Fig. S2). The dendogram is calculated from transformed parts per million (ppm) values that are used to calculate Spearman rank correlations between pairwise chaperone datasets (see Section 2 and Supporting Information >Table S2). Interestingly, the technically related approaches cluster together with epitope-tagging and MS-based methods found in two independent clades. Within the MS clade, the two label-mediated approaches show the highest correspondence, from our own studies and the SILAC study from de Godoy and colleagues 7. It is perhaps notable that different growth conditions do not produce apparent greater variation than different methodologies as shown by the close similarity between the GFP-tagging datasets for yeast grown in YPD and sucrose-deficient media 9. Naturally, some differences will also be expected between the yeast strains, as well as growth conditions. Our label-free (Q-Exactive Hi3 DDA) and label-mediated (QconCAT SRM) analyses also yield different chaperone cpc values despite being performed on identical yeast samples (strain and growth conditions), since they cluster to different regions of the dendogram. Harmonisation of the different quantification methods still seems to be out of reach.

**Figure 4:**
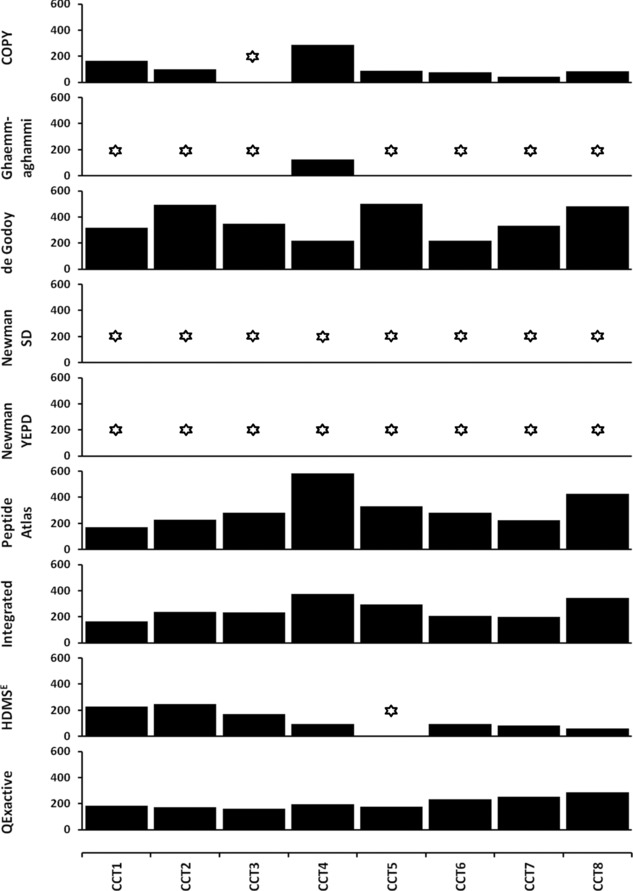
Protein quantification of the members of the CCT chaperone complex by alternative experimental strategies. The CCT complex is formed by an 8-mer of subunits forming a ring structure and is expected to retain a 1:1 stoichiometry between subunits. All protein abundance values are expressed as ppm for comparisons with PaxDB data, assuming 60 million copies in total for the QconCAT, Q-Exactive and HDMS^E^ datasets. The absence of measured quantification values is represented by a star symbol.

This comparison conceals some of the detail of the various studies, none of which were able to obtain values for all 63 yeast chaperones. Indeed as noted above, the CCT complex is poorly represented in the eptitope tagging studies 8,9 though it is present in most of the MS-based quantification studies. Figure 4 shows the eight major CCT proteins quantification values, expressed in ppm (for the purposes of this paper 1 ppm ≅ 60 cpc), extracted from the PaxDB database 26. Only Cct4 has been quantified by any of the epitope-tagging strategies to-date, while all of the MS-based methods have good success with all proteins in this complex. This is perhaps unsurprising since the CCT complex is formed by a heteromeric 8-mer ring structure which could be perturbed by any additional protein tagged on to it 29 and is believed to mediate the folding of a significant fraction of all cytosolic proteins 30. Furthermore, the CCT proteins are all classed as essential by saccharomyces genome database (SGD) 31,32 suggesting that techniques that perturb the structure or folding of such proteins could be lethal. It is also interesting to note that this class of chaperone shows the lowest CV of the abundance values across all the classes, given that it is an octamer with one:one stoichiometry 29. This result is independent of the method of quantification (see Supporting Information >Table S3). Comparing the quantifications across datasets, our most recent analysis using label-free methods on the Q-Exactive yields a stoichiometry closest to one:one for this chaperone class.

Finally, it is evident that the different quantitative approaches report different overall estimates of protein abundance, as epitomised by Fig. 4. Although it is difficult to directly compare the methods since they are converted to ppm values in PaxDB with somewhat arbitrary conversion factors, we can make direct comparisons of those reporting absolute quantitative values in cpc. In this case, the mean cpc values for all chaperones are 36 000, 52 000, 42 000 and 113 000 for the QconCAT, TAP-tagging, HDMS^E^ and Q-Exactive Hi3 label-free methods, respectively. The QconCAT data is lowest; this partly reflects the high sensitivity of this technique to report quantification via SRM at < 1000 cpc values for additional chaperones, lowering the overall average. For example, five of the HSP40 class of chaperones are reported at less than 500 cpc using our QconCATs but are absent from the HDSM^E^ and Q-Exactive Hi3 label-free quantifications entirely. It is also possible that epitope tagging can induce over-expression in some cases, which could lead to inflated cpc values.

### 3.3 Relationship between chaperone and target abundance

The chaperones mediate protein folding in the cell, regulating the stability and preventing mis-folding for a large component of the genome. Gong and colleagues 11 identified 4340 candidate substrates from their tandem affinity purification strategy that represents the majority of the yeast proteome. This qualitative map of the yeast chaperome network however lacks quantification, and here we add our own and published yeast protein abundance values to consider the folding ‘workload’ undertaken by each chaperone or its class. Additionally, given the tendency of affinity pull-down data to contain false positives, we have generated an additional ‘high-quality’ dataset of the chaperone interactome, comprising a total of 3649 interactions over 60 chaperones. All chaperone interactions were obtained from three public protein–protein interaction datasets (BioGRID 33, MIPS 34 and STRING 35). These were further filtered by excluding chaperone–chaperone interactions, retaining interactions where the reciprocal interaction was also observed (within a dataset) and in the case of STRING the interaction confidence score was >0.7 (high-confidence). The three filtered datasets were then combined to produce a ‘high-quality’ dataset, covering 60 chaperones, 1711 substrates and 3649 interactions. Analysis of the overlap between the datasets showed that MIPS provided no information additional to the combination of BioGRID and STRING (see Supporting Information Fig. S3A–C). The filtering process retained all but one of the complexes exhibiting reciprocity as reported by Gong and colleagues (see Supporting Information Fig. S3D).

**Figure 5:**
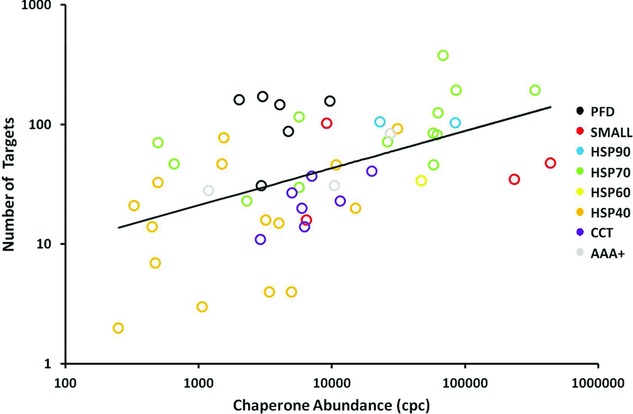
Number of chaperone-targets versus chaperone abundance. The number of non-chaperone substrates (or targets) is plotted against the abundance of each chaperone (in copies per cell units) for the QconCAT quantification dataset, against number of chaperone targets. A moderate correlation is observed with the more abundant chaperones having more interacting proteins (*R*_sp_: 0.49, *p* = 0.00002).

We next considered the relationship between chaperone abundance and the number of interactors, reasoning that chaperones with high numbers of targets ought to be abundant. Although no previous correlation had been noted 11, a modest but significant positive correlation is observed here (Fig. 5), for the high-quality filtered interaction set. This correlation is observed independently of the method used to quantify the chaperones, generating a Spearman Rank correlation statistic of 0.08–0.54 (see Supporting Information >Table S4). Simplistically, there is a correlation between the qualitative workload of a chaperone (as measured by the number of interactors) and its abundance in the cell. In this case, we have not included the chaperone–chaperone interactions as substrates since many will be co-chaperone interactions and do not strictly represent substrates. The correlation is also observed for the unfiltered complete set of interactions reported by Gong and colleagues 11, and some of the correlations observed are slightly stronger (Spearman Rank correlations between 0.48 and 0.74). Figure 5 also breaks down the chaperones into classes, showing that the generally low abundance HSP40 class have relatively few substrates while the generic ‘promiscuous’ chaperones with many substrates, such as the SMALL class and Hsp70 members (including Ssa1), are high in abundance.

These analyses only consider the number of interactors, not the total ‘volume’ of protein folding/translocation being mediated by a given chaperone. Accordingly, for each chaperone (c), the volume of protein (*V*_c_) was calculated as the total abundance (cpc_n_) of all *n* substrates.
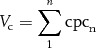


However, the volume does not directly consider the actual folding workload placed on chaperones which is influenced by rate of substrate turnover. To account for this, we use measured protein degradation rates *k*_deg_ 6 to estimate a synthesis rate, *k*_syn_, for individual chaperone targets, as shown below.
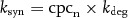


We can then sum these values to estimate the total workload or flux *F*_c_ (in molecules per minute) handled by an individual chaperone or chaperone class.
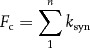


These values are based on some assumptions. We assume that protein abundances are in steady state (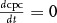) and the rate of synthesis captures the total flux dealt with by the attendant chaperones; we split flux on a pro rata basis across chaperones when there are multiple ‘parents’. Missing *k*_deg_ were substituted by the geometric mean across the entire dataset for instances where no turnover data was available for some substrates. We have also ignored growth rates, since our raw quantification and half-life data come from different sources of yeast experiment. Effectively, this adds a constant to *k*_deg_ values, akin to the dilution rate in a controlled culture system, which would alter our estimated fluxes. Nevertheless, we believe that despite the limitation of the data and assumptions, these values represent the most accurate current estimates of protein flux available that are, importantly, also comprehensive. We refer the interested reader to a review article that addresses several key issues and challenges in measuring protein turnover, which develops these ideas further 36.

Here, and for all further calculations, we use the filtered high-quality set of 3649 interactions rather than the complete set from Gong and colleagues 11. We do not have a genome-wide SRM-based quantitative dataset from QconCATs and therefore use the more comprehensive datasets from PaxDB and our own label-free data to capture substrate abundance.

**Figure 6:**
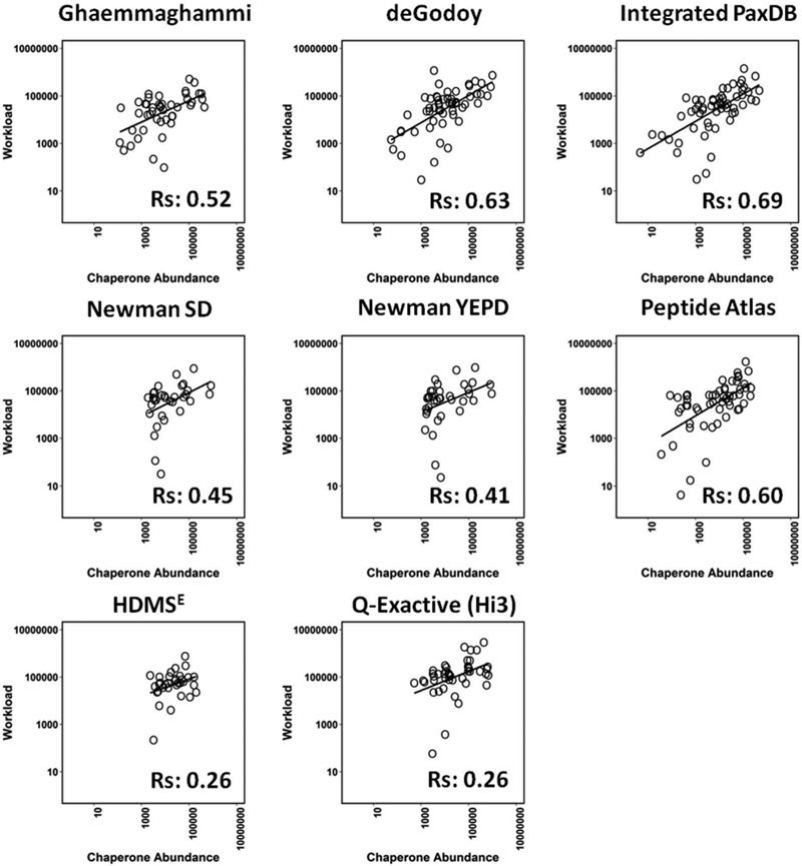
Analysis of chaperone abundance versus total target flux, *F*_c_. For each quantification dataset the chaperone protein abundances were plotted against the total flux *F*_c_ (workload) calculated from all its interacting proteins (where abundance values were obtained). Correlations are observed for each dataset, ranging from 0.26 to 0.69, which are all significant (*p* < 0.05) except the 0.26 obtained for HDMS^E^. For all but the Q-Exactive Hi3 and HDMS^E^ datasets, protein abundance data was taken from PaxDB 26, assuming 1 ppm = 60 cpc.

When considering either the total chaperone substrate abundance *V*_c_, or more formally, the total chaperone substrate flux *F*_c_, the correlation is maintained or strengthened. The Spearman correlation coefficients range from 0.26 to 0.69, for the comparison of chaperone abundance with flux as shown in Fig. 6. This demonstrates that chaperones mediating the folding of a large flux of expressed protein are themselves high-abundance proteins. The full set of both Pearson linear and Spearman Rank correlations are provided in Supporting Information >Table S4, which highlights that significant correlations are obtained for both volume and flux. We also see similar correlations when using the larger unfiltered protein interactions set (data not shown).

Figure 6 also clearly illustrates the reduced sensitivity of several label-free and epitope-tagging methodologies which have failed to quantify proteins (chaperones or substrates) below 1000 cpc, unlike the MS-based methods such as SILAC or the QconCAT approach presented here.

### 3.4 Chaperone workload and efficiency

As previously discussed, we can estimate the total chaperone ‘workload’ and the attendant ‘efficiency’ of individual chaperones and chaperone classes, defining the workload as the total substrate flux (*F*_c_). This definition, considers the cpc per minute estimated from the protein abundance and half-life as a proxy for the total amount of work a chaperone must undertake. Although this approach has some caveats, and does not directly factor in growth, it does provide a broad snapshot of the overall volume of protein folding meditated by individual chaperones. Table 2 shows the top 15 ranked chaperones by workload, calculated and ranked for the HQ filtered set of 3649 interactions. The proteins at the top of the list agree with expectation, containing ‘generalist’ chaperones that interact with significant fractions of nascent polypeptide chains close to or at the ribosome as part of the ribosomal-associated complex (RAC), or trafficking from Hsp70s and Hsp40s to Hsp90s. As would be expected, Table 2 is dominated by these chaperone classes, including RAC members Ssb1/2, other cytosolic Hsp70s including Ssa1, and nucleotide exchange factors such as Sse1 that are involved in trafficking onto the Hsp90 complex. It has been suggested that 20% of nascent polypeptide chains pass through the RAC/Hsp70/Hsp40 route 12 consistent with the high workload we observe here. Our estimate of over 1 million molecules per minute passing through the ribosome-associated Ssb1, suggests that each Ssb1 protein handles around 14 molecules per min. Similar statistics are observed when the unfiltered list of candidate substrates is used (Supporting Information >Table S5), though the estimated flux per chaperone increases to 25 molecules/min.

**Table 2:** Overall folding workload for top15 chaperones ranked by total substrate flux

Gene name	Systematic ORF name	Chaperone class	Chaperone abundance (cpc) a)	Number of substrates b)	Total Substrate volume (cpc) c)	Total substrate flux (cpc/min) d)
Ssb2	YNL209W	HSP70	85 650	172	13 675 400	1 172 700
Ssb1	YDL229W	HSP70	68 570	348	9 666 400	743 300
Ssz1	YHR064C	HSP70	63 090	115	6 167 800	423 800
Ssc1	YJR045C	HSP70	58 050	40	2 802 400	346 700
Ssa4	YER103W	HSP70	5700	29	2 261 500	323 800
Zuo1	YGR285C	HSP40	31 290	89	4 628 100	314 100
Hsc82	YMR186W	HSP90	84 370	90	3 853 900	274 600
Ssa1	YAL005C	HSP70	335 460	179	3 210 800	247 100
Cct2	YIL142W	CCT	7070	29	1 080 700	159 000
Cct4	YDL143W	CCT	20 010	32	1 044 700	150 000
Hsp60	YLR259C	HSP60	46 960	30	1 083 200	122 800
Sse1	YPL106C	HSP70	61 980	79	2 408 400	120 300
Hsp82	YPL240C	HSP90	23 040	89	1 338 500	103 000
Kar2	YJL034W	HSP70	26 300	66	935 100	96 300
Gim3	YNL153C	PFD	4060	120	1 162 300	95 400

a Chaperone cpc values were taken from our QconCAT yeast quantification.

b As determined from the high-quality filtered subset of 3649 interactions.

c Substrate abundance values were taken from PaxDB for the SILAC-based yeast quantification determined by de Godoy and colleagues 2008 and converted to cpc assuming 60 million protein molecules per cell.

d Flux was calculated as the sum over all chaperone substrates of the product of total substrate volume and the degradation rate, the latter obtained from the half-life study of Belle et al. 6.

**Figure 7:**
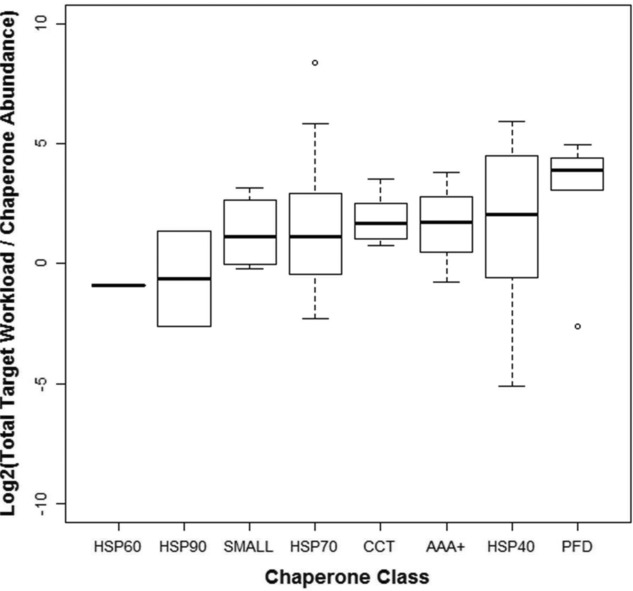
Relationship between chaperone abundance and normalised flux via each class. Box-and-whisker plots show the range of chaperone efficiency (flux/chaperone abundance) values calculated for individual chaperones in each class. The bold line shows the median value for each class, with boxes representing the inter-quartile ranges and whiskers the full extent of the minimum/maximum values in the normal way. The classes are ordered on median value from left to right. All workload efficiency values were calculated using the data from de Godoy and colleagues 7, normalising the total protein flux *F*_c_ by the chaperone cpc value, and then converting to a log_2_ value.

Table 2 raises the question as to whether there are chaperones (or chaperone groups) that are extremely (in)efficient in terms of the protein folding they mediate? Are there chaperones that despite being low abundance, mediate folding of a high-protein flux (high efficiency), or vice versa (low efficiency). This is an important concept, since not all chaperone proteins are expected to be directly and singly responsible for the folding of their targets 15, 37. To address this, the total substrate flux *F*_c_ can be normalised by dividing by the chaperone’s own abundance to calculate a workload efficiency. These are plotted as box-and-whisker plots in Fig. 7, on a log2 scale, grouped by chaperone class, using the PaxDB values from the de Godoy analysis to represent chaperone and target abundance 7. Very similar results are obtained when using any of the other comprehensive yeast protein abundance datasets (data not shown). A considerable variation is observed across the chaperones and their classes with apparently super-efficient chaperones such as Hsp70-Ssb2 (with log2 efficiency of >8) through to the apparently under-used chaperones such as the Hsp40-Djp1 (with a log efficiency of <−4). The latter is one of the regulatory J-domain chaperones which promotes the ATP-ase activity of Hsp70s and hence may not be directly interacting with true substrates; indeed, it is more abundant than the summed abundance of all its non-chaperone substrates. Interestingly, the prefoldins (PFD) as a class are the most efficient, an observation which is conserved irrespective of the quantification method used to calculate the values (Supporting Information >Fig. S4). These proteins form a heterohexameric complex that deliver nascent chains of TRiC/CCT substrates to this complex for refolding, including key structural proteins such as actin and tubulin.

**Figure 8:**
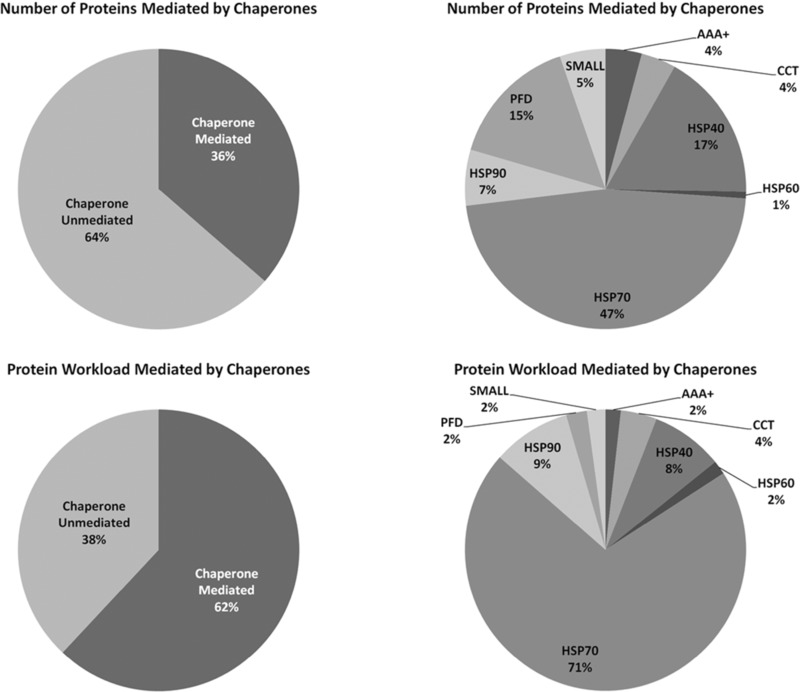
Overall chaperone workload in yeast. The two sets of pie charts consider the overall workload for chaperone classes in yeast, calculated by the number of proteins whose folding they mediate or scaled by the total protein flux for which they are responsible. In both cases, substrate counts or flux are shared pro rata between classes when more than one chaperone from different classes acts upon them. The two left most charts show how the amount of protein whose folding is regulated by chaperones in yeast is expanded when considered by total protein flux, and similar differences are observed when considered the breakdown of the data into chaperone classes on the right.

We can also consider the total volume and workload divided amongst the different chaperone classes, aggregating the abundance or flux of the targets to each class. If we assume that proteins not classed as chaperone targets can fold independently, we can consider the fraction of proteins (or protein abundance or flux) that is mediated by chaperone interactions in yeast (Fig. 8). Only 36% of proteins have been classed as chaperone targets but they constitute the majority (57%) of all protein volume in the cell, which itself represents 62% of the total flux of protein synthesis. Figure 8 also shows how the relative workload changes when we consider the flux weighted fraction compared to just the number of targets for each chaperone class (note: we share flux across classes pro rata when a substrate’s folding is mediated by more than one chaperone from different classes). This shows that although the Hsp70 class acts on fewer than 50% of known chaperone substrates, these proteins account for over 70% of the total chaperone-mediated protein synthesis flux in the cell. A similar small expansion is shown for the Hsp90 class while most others fall or stay roughly constant. Indeed, despite their efficiency and essentiality, the PFD and CCT chaperones mediate folding of less than 2 and 4%, respectively, of chaperone-mediated protein flux. The latter estimate is lower than previously reported values of ∼10% for CCT 30, 37, though we note our value is Iikely to be an underestimate given that we filter the interacting substrate set. Despite this, the CCT class in particular is enriched in targets which are themselves deemed essential (Supporting Information >Fig. S5). Around 50% of the protein abundance or flux regulated by CCT is annotated as ‘essential’ by the yeast genome deletion project 31, more than for any other chaperone class.

Integrating the abundance data with annotated essentiality, we can calculate how much of the yeast proteome is chaperone-mediated and deemed essential. The folding of approximately ∼9% of known ORFs are chaperone mediated and classed as essential, but this doubles to ∼18% when considered as a fraction of total protein flux, reinforcing the key role played by chaperones. Nevertheless, ∼16% of essential protein synthesis apparently requires no chaperone mediation.

Using the abundance estimates from different experimental approaches, we can estimate the total throughput of different chaperone classes with respect to the total protein in the cell (Supporting Information >Table S6). Around 6.5% of all protein flux passes through the PFD/CCT route, comparable to the previous estimates of ∼10% 12,30,38 in both yeast and bacterial systems. Similarly, around 7% of all protein folding is mediated by Hsp90s (a slightly lower estimate than the 20% previously reported). However, our data estimates that 44% of all protein synthesis passes through the Hsp70 class including that allied to the RAC, Hsp70, Hsp40 route, which suggests previously reported values of 20% may be under-estimates 38,39.

### 3.5 Chaperone target sub-cellular localisation

**Figure 9:**
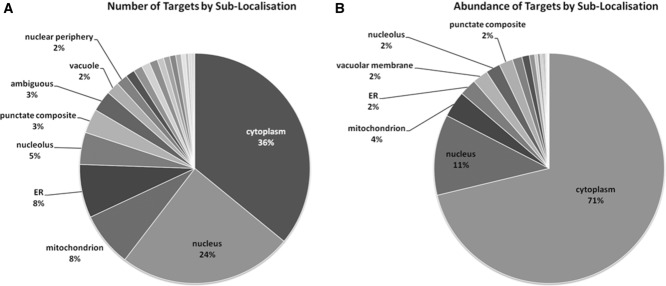
Sub-cellular disposition of the yeast proteome. The pie charts show the total proteome split into component sub-cellular localisations, annotating proteins from Huh et al. 5. In A, the number of proteins is used as a fraction of the total, while in B this is scaled by the total protein abundance. In both cases, proteins assigned to multiple loci and split pro rata between the different locations, either by count or by abundance. The differences between A and B highlight the expansions in abundance where the total concentration of protein assigned to a specific location exceed the relative number of proteins encoded in the genome. Sub-cellular locations containing less than 2% are not labelled for clarity, but also include ER to Golgi, Golgi to ER, Golgi to vacuole, actin, bud, bud neck, cell periphery, endosome, lipid particle, microtubule, peroxisome and spindle pole.

Since the sub-cellular localisation of the majority of yeast proteins has been characterised by a variety of studies, notably 5, we can scale the proteins in different locations by their abundance rather than just the number of different protein species. This is shown in Fig. 9 for one of the more comprehensive quantification datasets 7. Most of the cellular proteome is in the cytoplasm, as would be expected, followed by the membrane and mitochondrion, whether considered by proteins annotated in the genome or scaled by abundance. However, the cytoplasmic, membrane and mitochondrial protein abundances are expanded versus expectation (i.e. the total copy number of cytosolic proteins is larger than the total number annotated as such in the genome). Conversely, the nuclear protein abundance is reduced compared to expectation. This makes sense since despite the large number of proteins encoded in the genome destined for the nucleus, many of them will have signalling and regulatory roles (e.g. transcription factors) and would not be expected to be continually expressed or high abundance.

**Figure 10:**
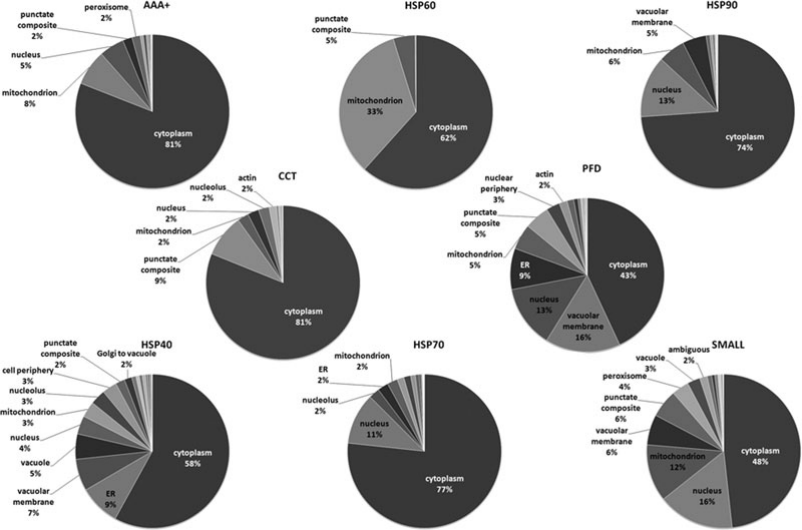
Abundance scaled sub-cellular proteomes of the different chaperone classes. The pie charts show the relative fraction of total protein volume of the sets of chaperone targets for each chaperone class, split by the sub-cellular localisation of the targets. The plots show that different chaperone classes display different preferences compared to the overall trends displayed in Fig. 8, specialising in the regulation of folding of proteins destined for different sub-cellular locations. Cytoplasmic proteins dominate all classes, but the SMALL class also has a preference towards nuclear and mitochondrial substrates, while prefoldins (PFD) are also linked to vacuolar membrane and ER, as well as of their key targets such as actin.

When the chaperone-mediated substrates are analysed by class, different distributions of substrate location emerge. The SMALL and PFD class both have quite different distributions to that for all proteins in Fig. 10, specialising in several non-cytosolic categories. Similarly, PFDtargets constitute a large fraction of protein destined for the vacuolar membrane, nucleus and ER. We also noted a small expansion in the mitochondrial class for Hsp90s, which concurs with the module assigned to Hsc82 by Bogumil and colleagues that also includes known mitochondrially active yeast chaperones 16. Indeed, this enrichment is statistically significant (*p* < 0.01) as are many of the individual values of fractional target protein volume in selected sub-cellular locations using the Expression Analysis Systematic Explorer (EASE) modified Fisher Exact statistical test 40. This includes the microtubular and mitochondrial targets for the PFD, ER, Golgi and nuclear targets for the Hsp40s and cytoplasm for the ‘generalist’ Hsp70s, all consistent with expectation in the literature (cf. 41,42). The full list of *p*-values for each sub-cellular location by chaperone class are listed in Supporting Information >Table S7.

The data are consistent with a degree of specialisation for different chaperone classes in terms of the sub-cellular destination of their targets, notwithstanding the fact that the majority of their targets are normally cytosolic. Indeed, the observed sub-cellular specialisation by target protein volume is also statistically significant across the chaperone classes (*p* < 0.001, using Kruskal–Wallis), an observation that is clear when the data are viewed from the perspective of the sub-cellular localisations (Supporting Information >Figs. S6 and >S7) which highlights, for example, the PFD/CCT preference for actins and microtubule proteins such as tubulin.

No clear specialisations for substrate location were reported in a previous comprehensive study although a different approach was taken to the assignment of chaperones to modules and quantification was not directly factored in 16.

## 4 Concluding remarks

We present here, for the first time, absolute quantification of a functional class of the yeast proteome using the QconCAT methodology as part of our COPY project. We have successfully quantified 51 of the 63 annotated chaperones in the yeast proteome by SRM using stable isotope labelled surrogate peptides with high precision, achieving quantification down to *ca*. 250 cpc. The sibling peptides for individual proteins show a high level of agreement and we believe this represents the most accurate measurement of chaperone abundance in yeast. Nevertheless, the comparison with other approaches highlights the disparity that still exists in quantitative proteomics and the variance introduced by the different methodologies, which apparently exceeds that occurring biologically.

We elected in most cases to use the SILAC-based quantification data 7 to present aggregated abundance data for protein volume, flux, folding efficiency and sub-cellular localisation. This is one of the most comprehensive studies, with over 4000 proteins quantified. We reasoned that the relatively small number of proteins missed by this set are likely to be low abundance or not expressed, and therefore cannot greatly skew the analysis. Moreover, this is an MS-based dataset that does not involve epitope tagging (which could on occasions lead to lethal fusions or over-expression). Nevertheless, the global trends are maintained, regardless of dataset used to make the calculations (data not shown).

The availability of quantitative datasets such as these supports the estimation of protein synthetic flux (workload) and associated efficiency, which coupled to interactome and half-life data allows the different chaperones and classes to be compared. We report for the first time the direct correlation between chaperone abundance and the number, aggregated abundance and flux of their targets, an observation which makes energetic sense for yeast cells. This extends other reported data; for example, Bogumil et al. 16 note that AAA+ and CCT chaperones substrates are highly expressed and have high-codon adaptation indices, as do the chaperone modules which contain the chaperones themselves. We see this trend extended broadly across all classes, independent of experimental approach to quantify the proteome.

We also see evidence that yeast chaperones are biased to mediate the folding of substrates from different sub-cellular localisations. Although the comprehensive study by Bogumil and colleagues 16 highlighted a broad spread of sub-cellular locations within chaperone modules they did not observe specific module trends. We confirm the observation that chaperones appear to mediate folding across a broad range of different sub-cellular localisations, and not just the one(s) where they are believed to reside.

The integrated interactome and quantification data offer an alternative method to estimate chaperone substrate throughput, which may be compare to previous studies. Our analyses provide new estimates on the volume of protein folding passing for through the different chaperone pathways and classes, and we estimate as much as 44% of all protein synthesis goes through the RAC-induced route via Hsp70s and Hsp40s.

Quantitative proteomics data, especially on absolute concentrations, can provide a new interpretation of affinity pull-down inter-actomics data and better characterise molecular systems such as the chaperone network. We aim to extend these generalised findings to develop our understanding of the chaperone network and the dynamics and stoichiometry of protein folding and proteostasis in yeast.

## Abbreviations

Cpc: copies per cell
PFD: prefoldins
RAC: ribosomal-associated complex
SGD: Saccharomyces Genome Database
TAP: Tandem Affinity Purification
XICs: extracted ion chromatograms

## Notes

As a service to our authors and readers, this journal provides supporting information supplied by the authors. Such materials are peer reviewed and may be re-organized for online delivery, but are not copy-edited or typeset. Technical support issues arising from supporting information (other than missing files) should be addressed to the authors.

**Table S1** Complete set of copies per cell quantitation values obtained for the chaperone proteins **Table S2** Spearman Rank correlation matrix between all quantification methods for the chaperone dataset. **Table S3** Chaperone quantitation statistics from different studies, including Coefficient of Variation (CV) statistics **Table S4** Chaperone and substrate count correlation statistics. **Table S5** Overall folding for top15 chaperones as determined using Gong and colleagues protein-protein interaction data. **Table S6** Overall distribution of chaperone-mediated and independent folding for all yeast proteins. **Table S7** Significance testing of total abundance of protein substrate folding mediated by chaperone class. **Figure S1A** Light and heavy peptide transition XICs Peptide DVSVEEDISELLR for P40358. **Figure S1B** Light and heavy peptide transition XICs for Peptide NTINEASFK for P09435. **Figure S2** Scatter plot matrix comparing all chaperone abundances across the different quantification methods. **Figure S3** Overlap between Protein-Protein interaction datasets used in this study. **Figure S4** Chaperone workload efficiency box-and-whisker plots. **Figure S5** Fraction of chaperone class target abundance classed as essential **Figure S6** Proportion of protein abundance of sub-cellular localisation by chaperone mediation and non-mediation. **Figure S7** Proportion of protein abundance of chaperone mediated sub-cellular localisation by chaperone classes.
