# Supplementary material for: Quantitative analysis of chaperone network throughput in budding yeast
Source: Proteomics. 2013 Mar 15;13(8):1276–91. doi: 10.1002/pmic.201200412 (PMC3791555; doi:10.1002/pmic.201200412)

**Supplementary material**

Quantitative analysis of chaperone network throughput in budding yeast

Philip Brownridge, Craig Lawless, Aishwarya B. Payapilly, Karin Lanthaler, Stephen W. Holman, Victoria M. Harman, Christopher M. Grant, Robert J. Beynon, Simon J. Hubbard

Supplementary Methods 1

Further details of mass spectrometry 1

mQuest parameters used as part of the mProphet pipeline. 3

Table S1. Complete set of copies per cell quantitation values obtained for the chaperone proteins 4

Table S2. Spearman Rank correlation matrix between all quantification methods for the chaperone dataset. 7

Table S3. Chaperone quantitation statistics from different studies, including Coefficient of Variation (CV) statistics 8

Table S4. Chaperone and substrate count correlation statistics. 10

Table S5. Overall folding for top15 chaperones as determined using Gong and colleagues protein-protein interaction data. 10

Table S6. Overall distribution of chaperone-mediated and independent folding for all yeast proteins. 11

Table S7. Significance testing of total abundance of protein substrate folding mediated by chaperone class. 12

Figure S1.A. Light and heavy peptide transition XICs Peptide DVSVEEDISELLR for P40358 . 14

Figure S1B. Light and heavy peptide transition XICs for Peptide NTINEASFK for P09435. 15

Figure S2. Scatter plot matrix comparing all chaperone abundances across the different quantification methods. 16

Figure S3. Overlap between Protein-Protein interaction datasets used in this study. 17

Figure S4. Chaperone workload efficiency box-and-whisker plots. 18

Figure S5. Fraction of chaperone class target abundance classed as essential 19

Figure S6. Proportion of protein abundance of sub-cellular localisation by chaperone mediation and non-mediation . 20

Figure S7. Proportion of protein abundance of chaperone mediated sub-cellular localisation by chaperone classes. 21

## Supplementary Methods

### Further details of mass spectrometry

Each tryptic digest was analysed by LCMS using a nanoAcquity UPLC™ system (Waters) coupled to a Synapt™ G2 mass spectrometer (Waters) to verify complete digestion and to quantify the CopyCAT. The sample (1 µL corresponding to approximately the protein equivalent of 100,000 cells) was loaded onto the trapping column (Waters C18, 180 μm X 20 mm), using partial loop injection, for three minutes at a flow rate of 5 μL/min with 0.1% (v/v) TFA. The sample was resolved on an analytical column (nanoACQUITY UPLC™ HSS T3 C18 75 µm x 150 mm 1.7 µm column) using a gradient of 97% A (0.1% formic acid) 3% B (99.9% ACN 0.1% formic acid) to 60% A 40% B over 60 minutes at a flow rate of 300 nL min^-1^. The mass spectrometer acquired data using a data independent program with 1 s scan times and a collision energy ramp of 15 to 40 eV for elevated energy scans. The data was processed and database searched using ProteinLynx Global Server v2.5 (Waters). The data was processed using a low energy threshold of 100 and an elevated energy threshold of 20 and the processed spectra were searched against a database created from the sequences of CopyCAT 1, 2 and 3 using fixed modifications for carbamidomethyl modification of cysteine and ^13^C_6_ modification of arginine and lysine.

SRM analysis was performed using a nanoAcquity UPLC™ system (Waters) (as described earlier in the main paper) coupled to a Xevo™ TQ triple quadrupolar mass spectrometer (Waters) The mass spectrometer was operated in scheduled SRM mode with Q1 and Q3 operating at unit resolution. The program was set to acquire 15 datapoints over a 30 s chromatographic peak within a 4 minute window. The final transition list, based on the three highest performing transitions, was divided in two to achieve a minimum dwell time of 50ms and each sample analysed with both transition lists.

The data-dependent label-free analysis was performed using an Ultimate 3000 RSLC™ nano system (Thermo Scientific) coupled to a QExactive™ mass spectrometer (Thermo Scientific). The sample (5 µL corresponding to 400,000 cells and 50fmol glycogen phosphorylase) was loaded onto the trapping column (Thermo Scientific, PepMap100, C18, 75 μm X 20 mm), using partial loop injection, for seven minutes at a flow rate of 4 μL/min with 0.1% (v/v) FA. The sample was resolved on the analytical column (Easy-Spray C18 75 µm x 500 mm 2 µm column) using a gradient of 97% A (0.1% formic acid) 3% B (99.9% ACN 0.1% formic acid) to 60% A 40% B over 240 minutes at a flow rate of 300 nL min^-1^. The data-dependent program used for data acquisition consisted of a 70,000 resolution full-scan MS scan (AGC set to 1e6 ions with a maximum fill time of 250ms) the 10 most abundant peaks were selected for MS/MS using a 17,000 resolution scan (AGC set to 5e4 ions with a maximum fill time of 250ms) with an ion selection window of 3 *m/z* and a normalised collision energy of 30. To avoid repeated selection of peptides for MSMS the program used a 30 second dynamic exclusion window.

Database searching was performed using MASCOT (Matrix Science). A Mascot Generic File was created by Progenesis and searched against the reference proteome set of *S.cerevisiae* from Uniprot (6560 proteins) with the sequence of rabbit glycogen phosphorylase (UniProt: P00489) added. A fixed carbamidomethyl modification for cysteine and variable oxidation modification for methionine were specified. A precursor mass tolerance of 10 ppm and a fragment ion mass tolerance of 20 mmu were applied. At a p value of 0.05, the built in MASCOT decoy search reported a peptide FDR of 0.5%.

### mQuest parameters used as part of the mProphet pipeline.

Params.def file optimised for use with mQuest.pl program of the mProphet package.

use_reference 1

allow_pgpair_wo_reference_partner 0

allow_pgpair_wo_target_partner 1

make_dummy_peakgroup 1

fill_pgs_with_noise 1

light_label light

heavy_label heavy

reference_isoform heavy

minSN_target 5

minSN_reference 5

select_nbest_peakgroups_target 4

select_nbest_peakgroups_reference 2

use_decoy 1

decoy_schema AQUA

main_vars log10_total_xic|intensity_correlation_with_assay|xcorr_coelution_score|xcorr_shape_score|light_heavy_correlation|light_heavy_shape_score|light_heavy_coelution_score|abs_Tr_deviation

main_score light_heavy_shape_score

min_peak_width 7

select_nbest_peaks 5

max_Tr_difference 5

denoise_parameter1 5

## Table S1. Complete set of copies per cell quantitation values obtained for the chaperone proteins

| **Chaperone Class** | **SGD Gene Name** | **UniProt** | **Peptide** | **Bio-Rep** | | | | **Peptide CpC** |
| --- | --- | --- | --- | --- | --- | --- | --- | --- |
|  |  |  |  | **1** | **2** | **3** | **4** |  |
| SMALL | Hsp12 | P22943 | ASEALKPDSQK | 466154.7 | 389644.3 | 373917 | 525767 | 438870.7758 |
|  |  |  | LNDAVEYVSGR | 349612.7 | 288453.9 | 255757.7 | 354722.4 | 312136.6862 |
| SMALL | Hsp26 | P15992 | DIDIEYHQNK | 310721.5 | 266855.4 | 199739.7 | 162019.6 | 234834.0313 |
|  |  |  | VITLPDYPGVDADNIK | 243837.8 | 229564.8 | 149281.1 | 131036 | 188429.9279 |
| SMALL | Hsp31 | Q04432 | LVTGVNPASAHSTAVR | 6938.455 | 5942.891 | 5152.38 | 7775.072 | 6452.199456 |
| SMALL | Hsp42 | Q12329 | GQQGYPR | 9988.992 | 9211.874 | 9550.334 | 7947.058 | 9174.564535 |
| PFD | Gim3 | P53900 | VTAQLEK | 4272.74 | 3954.96 | 3719.884 | 3736.351 | 3920.983627 |
|  |  |  | NNTQVTFEDQQK | 4877.64 | 4109.248 | 3171.211 | 4075.886 | 4058.496384 |
| PFD | Gim4 | P40005 | NNVFQAK | 10337.75 | 9260.612 | 9036.314 | 10033.92 | 9667.150914 |
| PFD | Gim5 | Q04493 | LLVPASASLYIPGK | 2239.149 | 1854.903 | 2014.455 | 1973.051 | 2020.389501 |
| PFD | Pac10 | P48363 | AQVDIPEDR | 2746.894 | 2661.604 | 2996.45 | 2664.383 | 2767.332806 |
|  |  |  | IPDLENTLK | 4747.952 | 4407.7 | 5322.408 | 4456.783 | 4733.710591 |
| PFD | Pfd1 | P46988 | SFILQDK | 3156.41 | 2861.606 | 2506.412 | 3340.014 | 2966.11043 |
|  |  |  | NYLETTVEK | 1373.294 | 1451.634 | 1131.653 | 1402.43 | 1339.75259 |
| PFD | Yke2 | P52553 | LEFIETEITR | 3228.415 | 3552.053 | 3209.497 | 2110.587 | 3025.137861 |
|  |  |  | LTGNVLLPVEQSEAR | 2383.07 | 2229.627 | 1358.19 | 1154.123 | 1781.252619 |
| HSP90 | Hsc82 | P15108 | DILGDQVEK | 88926.26 | 86969.34 | 81632.14 | 79962.94 | 84372.66791 |
| HSP90 | Hsp82 | P02829 | EILGDQVEK | 24745.33 | 23190.52 | 23193.39 | 21025.17 | 23038.60347 |
| HSP70 | Kar2 | P16474 | ITPSYVAFTDDER | 26499.27 | 25929.21 | 23303.6 | 29455.04 | 26296.78252 |
| HSP70 | Lhs1 | P36016 | SLEDETTVTLYSK | 2942.886 | 1836.257 | 2529.851 | -- | 2436.331086 |
| HSP70 | Ssa1 | P10591 | TTPSFVAFTDTER | 403105.9 | 339949.5 | 281593.9 | 317189.2 | 335459.6148 |
|  |  |  | AEETISWLDSNTTASK | 65333.78 | 77080.89 | 47586.52 | 32742.32 | 55685.876 |
| HSP70 | Ssa2 | P10592 | EEFDDQLK | 54176.71 | 50949.51 | 62941.55 | 59822.04 | 56972.45384 |
|  |  |  | TTPSFVGFTDTER | 51056.43 | 51011.55 | 71830.03 | 57101.77 | 57749.94452 |

| HSP70 | Ssa3 | P09435 | NTINEASFK | 722.7888 | 605.4043 | 531.7897 | 773.6898 | 658.4181412 |
| --- | --- | --- | --- | --- | --- | --- | --- | --- |
| HSP70 | Ssa4 | P22202 | ATAGDTHLGGEDFDSR | 1841.868 | 1599.934 | 1201.977 | 2118.219 | 1690.499362 |
|  |  |  | DAVVTVPAYFNDSQR | 5800.1 | 5652.918 | 5621.532 | 5743.932 | 5704.620348 |
| HSP70 | Ssb1 | P11484 | VTPSFVAFTPEER | 65657.67 | 66581.79 | 69431.3 | 72604.62 | 68568.84418 |
| HSP70 | Ssb2 | P40150 | TFTTVSDNQTTVQFPVYQGER | 59932.04 | 47271.41 | 35910.98 | 46408.85 | 47380.82102 |
|  |  |  | VTPSFVAFTPQER | 88941.18 | 80789.99 | 79649.55 | 93225.19 | 85651.47646 |
| HSP70 | Ssc1 | P12398 | VVNEPTAAALAYGLEK | 69249.76 | 53128.13 | 54367.26 | 55443.28 | 58047.10846 |
| HSP70 | Sse1 | P32589 | AEEWLYDEGFDSIK | 14629.22 | 13212.65 | 12181.17 | 13229.97 | 13313.25116 |
|  |  |  | IVNDVTAAGVSYGIFK | 63735.54 | 58474.09 | 66591.63 | 59135.29 | 61984.13606 |
| HSP70 | Sse2 | P32590 | IVNDVTAAAVSYGVFK | 5664.378 | 5234.481 | 6345.959 | 5530.063 | 5693.720161 |
|  |  |  | VGVEVEFGGK | 7042.104 | 5554.767 | 4316.229 | 5916.389 | 5707.372271 |
| HSP70 | Ssq1 | Q05931 | SATIIENDEGQR | 2521.834 | 2312.678 | 2044.823 | 2328.835 | 2302.042373 |
| HSP70 | Ssz1 | P38788 | LAAEDYIGSAVK | 7712.567 | 6847.658 | 7512.845 | 8060.692 | 7533.440394 |
|  |  |  | NDVDVIANPDGER | 62730.23 | 56962.06 | 73535.65 | 59135.49 | 63090.85522 |
| HSP60 | Hsp60 | P19882 | AAVEEGILPGGGTALVK | 54378.54 | 44794.27 | 45146.34 | 43532.79 | 46962.98519 |
|  |  |  | GVETLAEAVAATLGPK | 46069 | 40632.7 | 43205.91 | -- | 43302.53623 |
| HSP40 | Caj1 | P39101 | SAYNLLSTGLEAQK | 5847.739 | 4907.493 | 4288.953 | 4928.514 | 4993.174562 |
| HSP40 | Djp1 | P40564 | VLEDDSVSK | 535.6134 | 450.2578 | 519.2256 | 529.1038 | 508.5501421 |
|  |  |  | VLSAAWHGSK | 1217.872 | 1284.096 | 906.3588 | 837.8477 | 1061.543611 |
| HSP40 | Erj5 | P43613 | IENFISQCK | 3470.406 | 3310.3 | 3152.012 | 3666.033 | 3399.687656 |
| HSP40 | Jac1 | P53193 | YWYNLAK | 481.2504 | 528.4291 | 423.1119 | 463.7275 | 474.1297208 |
| HSP40 | Jem1 | P40358 | DVSVEEDISELLR | 369.0344 | 404.8743 | 533.5649 | 483.9232 | 447.8492255 |
| HSP40 | Jjj1 | P53863 | IDNSAAGIYQIAGK | 697.6692 | 395.3235 | 442.9926 | 323.2803 | 464.8163715 |
|  |  |  | SNELDDLLASLGDK | 445.1641 | 647.3625 | 568.8029 | 316.7291 | 494.5146493 |
| HSP40 | Mdj1 | P35191 | VSCSTCHGTGTTVHIR | 2796.733 | 3460.706 | 2894.501 | 3674.77 | 3206.677639 |
| HSP40 | Mdj2 | P42834 | IENPTAGYR | 306.5761 | 170.5962 | 370.5031 | 154.0522 | 250.4319075 |
|  |  |  | TLSPLTIAK | 222.0687 | 191.1314 | 279.3159 | 242.1559 | 233.6679762 |
| HSP40 | Scj1 | P25303 | IYDQFGADAVK | 1315.276 | 1804.345 | 1498.021 | 1388.3 | 1501.485801 |
| HSP40 | Sec63 | P14906 | AYESLTDELVR | 5381.328 | 5032.652 | 5102.244 | 4245.329 | 4940.388073 |
|  |  |  | FDINDWEIGTIK | 11099.45 | 9665.593 | 10233.75 | 12069.05 | 10766.96003 |

| HSP40 | Sis1 | P25294 | EIYDQYGLEAAR | 15984.02 | 14761.65 | 13655.73 | 15803.14 | 15051.13479 |
| --- | --- | --- | --- | --- | --- | --- | --- | --- |
|  |  |  | DGDDLIYTLPLSFK | 10862.2 | 9802.856 | 11047.94 | 13508 | 11305.25187 |
| HSP40 | Swa2 | Q06677 | VDFSAPPLVPTNSTTK | 1463.159 | 1596.085 | 1639.711 | 1503.9 | 1550.713733 |
| HSP40 | Tim14 | Q07914 | EALQILNLTENTLTK | 3936.01 | 4238.305 | 3696.313 | 4179.018 | 4012.411663 |
| HSP40 | Xdj1 | P39102 | FGPGLVASQWVVCEK | 343.6442 | 371.4851 | 309.108 | 284.403 | 327.1600686 |
| HSP40 | Zuo1 | P32527 | ATESQIIK | 24498.31 | 22669.81 | 23630.88 | 23233.45 | 23508.11123 |
|  |  |  | EADYFGDADK | 32456.38 | 28753.43 | 34578.96 | 29389.71 | 31294.61926 |
| CCT | Cct2 | P39076 | AVVSSASEAAEVLLR | 7918.502 | 7266.513 | 6030.991 |  | 7072.002327 |
|  |  |  | LASAAALDALTK | 5011.122 | 4968.459 | 3873.932 | 3952.569 | 4451.520448 |
| CCT | Cct4 | P39078 | GEIIISNDGHTILK | 22000.18 | 17196.91 | 20704.03 | 20136.01 | 20009.28179 |
|  |  |  | LGSADLVEEIDSDGSK | 5751.257 | 5364.927 | 5893.347 | 4643.431 | 5413.240852 |
| CCT | Cct5 | P40413 | IYEQEFGTTK | 6079.809 | 6300.582 | 6018.129 | 6709.195 | 6276.928787 |
| CCT | Cct6 | P39079 | GLVLDHGGR | 4083.026 | 4376.354 | 8009.906 | 4749.735 | 5304.7552 |
| CCT | Cct7 | P42943 | GGAEQVIAEVER | 1796.297 | 1425.732 | 2146.62 |  | 1789.549703 |
|  |  |  | LPIGDLATQFFADR | 3092.054 | 2829.796 | 2798.928 |  | 2906.926166 |
| CCT | Cct8 | P47079 | LLPGAGATEIELISR | 5217.69 | 5185.129 | 5300.031 | 4905.514 | 5152.090873 |
|  |  |  | LGAPTPEELGLVETVK | 5928.425 | 5811.989 | 5879.554 | 6290.522 | 5977.622713 |
| CCT | Tcp1 | P12612 | IIDAGAQVVLTTK | 13812.84 | 12860.42 | 11194.31 | 8310.492 | 11544.51452 |
| AAA+ | Hsp104 | P31539 | IIDDDVPTILQGAK | 28328.95 | 27102.65 | 23743.2 | 21760.79 | 25233.89672 |
|  |  |  | SNPCLIGEPGIGK | 31595.74 | 28785.47 | 26249.81 | 23108.96 | 27434.99827 |
| AAA+ | Hsp78 | P33416 | AIDLVDEACAVLR | 9361.082 | 10985.23 | 9557.983 | 11935.53 | 10459.95597 |
| AAA+ | Mcx1 | P38323 | SNVLVVGPSGSGK | 1054.725 | 1085.643 | 933.3089 | 1180.031 | 1063.426675 |
|  |  |  | DVSGEGVQQSLLK | 1283.591 | 1843.421 | 605.1456 | 1013.702 | 1186.46485 |

**
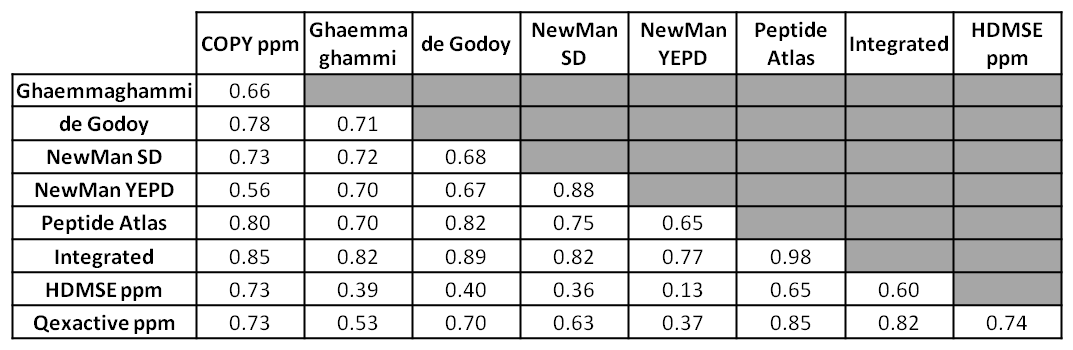
**

## Table S2. Spearman Rank correlation matrix between all quantification methods for the chaperone dataset.

## Table S3. Chaperone quantitation statistics from different studies, including Coefficient of Variation (CV) statistics

| **Chaperone Complex** | **Systematic Name** | **UniProt** | **Copy CPC** | **Ghaemmaghammi CPC** | **de Godoy ppm** | **Newman SD ppm** | **Newman YEPD ppm** | **Peptide Atlas ppm** | **Integrated Paxdb ppm** | **HDMSE CPC** | **Q-Exactive CPC** | **Avg. %CV** |
| --- | --- | --- | --- | --- | --- | --- | --- | --- | --- | --- | --- | --- |
| SMALL | YFL014W | P22943 | 438870.8 | 4485.1 | 357.5 | 342.0 | 35.2 | 1143.7 | 750.0 | 162891.0 | 679585.2 |  |
| SMALL | YBR072W | P15992 | 234834.0 | 19342.6 | 94.9 | - | 27.5 | 1004.4 | 594.1 | 200318.2 | 573803.8 |  |
| SMALL | YDR171W | Q12329 | 9174.6 | 1471.6 | 93.2 | 119.0 | 77.5 | 68.3 | 78.1 | 6218.5 | 14569.1 |  |
| SMALL | YDR533C | Q04432 | 6452.2 | 358.0 | 134.5 | 75.5 | - | 138.1 | 113.1 | 5623.1 | 26516.6 |  |
| SMALL | YMR322C | Q04902 | - | - | - | - | - | - | - | - | 56.2 |  |
| SMALL | YOR391C | Q08914 | - | - | - | - | - | - | - | - | - |  |
| SMALL | YPL280W | Q08992 | - | - | - | - | - | - | - | - | - |  |
|  |  | **CV** | **120.4** | **137.1** | **74.4** | **80.0** | **57.6** | **95.8** | **88.4** | **109.4** | **130.5** | **99.3** |
| PFD | YEL003W | P40005 | 9667.2 | 1655.9 | 80.5 | 31.5 | 36.6 | 7.0 | 29.5 | - | - |  |
| PFD | YGR078C | P48363 | 4733.7 | 6547.8 | 89.2 | 123.8 | 114.4 | 315.5 | 222.2 | - | 3530.8 |  |
| PFD | YNL153C | P53900 | 4058.5 | 7474.4 | 75.6 | 411.2 | 272.8 | 7.6 | 90.2 | - | 3136.6 |  |
| PFD | YLR200W | P52553 | 3025.1 | 783.8 | 42.4 | 70.4 | 108.6 | 116.4 | 92.5 | - | 5283.9 |  |
| PFD | YJL179W | P46988 | 2966.1 | 720.5 | 107.1 | 58.5 | 52.1 | 9.7 | 40.3 | - | - |  |
| PFD | YML094W | Q04493 | 2020.4 | 2903.6 | 144.2 | 56.5 | 48.7 | 86.3 | 91.9 | - | 3208.5 |  |
|  |  | **CV** | **62.1** | **88.4** | **37.9** | **114.4** | **83.6** | **132.5** | **72.6** |  | **26.7** | **77.3** |
| HSP90 | YMR186W | P15108 | 84372.7 | 132053.4 | 1809.0 | 58.3 | 102.5 | 1916.5 | 1564.4 | 93737.5 | 104371.7 |  |
| HSP90 | YPL240C | P02829 | 23038.6 | 444942.7 | 10433.3 | 1901.6 | 2708.8 | 1160.4 | 3697.0 | 34737.2 | 301195.3 |  |
|  |  |  |  |  |  |  |  |  |  |  |  |  |
| HSP70 | YAL005C | P10591 | 335459.6 | 268716.5 | 14403.0 | 14053.2 | 13721.8 | 3639.5 | 8084.0 | 163713.7 | 641049.0 |  |
| HSP70 | YNL209W | P40150 | 85651.5 | 103902.0 | 59.0 | 2636.2 | 3134.7 | 2150.7 | 1840.0 | 68684.8 | 67106.9 |  |
| HSP70 | YDL229W | P11484 | 68568.8 | 170038.6 | 16876.2 | 495.6 | 515.9 | 2845.8 | 5534.7 | 71896.0 | 442565.1 |  |
| HSP70 | YHR064C | P38788 | 63090.9 | 73590.4 | 3179.9 | 842.8 | 1109.1 | 983.8 | 1492.0 | 26998.3 | 89725.5 |  |
| HSP70 | YPL106C | P32589 | 61984.1 | 71727.1 | 5466.1 | 975.6 | 2446.8 | 2164.5 | 2814.6 | 58468.7 | 99594.6 |  |
| HSP70 | YJR045C | P12398 | 58047.1 | - | 5881.7 | - | - | 907.7 | 1811.3 | 41597.8 | 123234.3 |  |
| HSP70 | YLL024C | P10592 | 57749.9 | 364127.7 | 4115.1 | 12350.9 | 15773.8 | 3435.8 | 5829.7 | 42885.2 | 590165.4 |  |
| HSP70 | YJL034W | P16474 | 26296.8 | 336941.9 | 1941.3 | 86.0 | 83.4 | 451.5 | 906.4 | 26100.1 | 113549.0 |  |
| HSP70 | YBR169C | P32590 | 5707.4 | 6301.7 | 94.8 | 154.0 | 61.0 | 247.7 | 182.2 | 27390.9 | 95961.1 |  |
| HSP70 | YER103W | P22202 | 5704.6 | 17876.6 | 95.0 | - | 66.3 | 1081.8 | 640.5 | 2342.1 | 224022.3 |  |
| HSP70 | YLR369W | Q05931 | 2302.0 | 5547.8 | 28.5 | 52.5 | 36.4 | 7.5 | 22.3 | - | 1244.7 |  |
| HSP70 | YKL073W | P36016 | 2436.3 | 136.8 | 109.7 | - | - | 18.3 | 34.6 | - | 3535.5 |  |
| HSP70 | YBL075C | P09435 | 658.4 | 6442.0 | - | - | - | 664.8 | 373.3 | 109728.7 | 545368.1 |  |
| HSP70 | YEL030W | P39987 | - | - | 141.5 | - | - | 173.9 | 128.0 | 47045.5 | 77255.6 |  |
|  |  | **CV** | **148.6** | **113.4** | **139.0** | **158.2** | **160.8** | **94.8** | **120.7** | **75.9** | **102.5** | **123.8** |
| HSP60 | YLR259C | P19882 | 46963.0 | - | 3845.0 | - | - | 1257.5 | 1553.0 | 51400.4 | 94750.3 |  |
|  |  |  |  |  |  |  |  |  |  |  |  |  |
| HSP40 | YGR285C | P32527 | 31294.6 | 86409.0 | 1739.8 | 1488.9 | 1653.6 | 1028.5 | 1319.1 | 17311.1 | 113898.6 |  |
| HSP40 | YNL007C | P25294 | 15051.1 | 20306.1 | 655.7 | 711.8 | 717.5 | 323.9 | 477.2 | 17256.4 | 37858.8 |  |
| HSP40 | YOR254C | P14906 | 10767.0 | 17716.0 | 381.9 | 267.1 | 318.7 | 62.9 | 188.4 | 4613.4 | 8843.0 |  |
| HSP40 | YER048C | P39101 | 4993.2 | - | 227.3 | - | - | 46.4 | 76.3 | 3248.4 | 10390.2 |  |
| HSP40 | YLR008C | Q07914 | 4012.4 | - | 39.3 | - | - | 9.6 | 14.0 | - | 5666.5 |  |
| HSP40 | YFR041C | P43613 | 3399.7 | - | 17.2 | 63.7 | 66.9 | 4.1 | 18.8 | - | 3035.5 |  |
| HSP40 | YFL016C | P35191 | 3206.7 | 7572.9 | 55.4 | 150.5 | 85.4 | 37.5 | 59.8 | - | 3364.8 |  |
| HSP40 | YDR320C | Q06677 | 1550.7 | 768.1 | 24.4 | 52.7 | 50.2 | 3.0 | 17.5 | - | - |  |
| HSP40 | YMR214W | P25303 | 1501.5 | 8260.3 | 60.1 | - | 31.6 | 21.2 | 33.8 | - | 1388.1 |  |
| HSP40 | YIR004W | P40564 | 1061.5 | 8965.9 | 59.9 | 111.3 | 113.3 | 10.0 | 46.3 | - | 10186.4 |  |
| HSP40 | YNL227C | P53863 | 494.5 | 2313.3 | 2.4 | - | - | 4.0 | 4.2 | - | - |  |
| HSP40 | YGL018C | P53193 | 474.1 | 2010.0 | 9.0 | 44.8 | - | 7.7 | 11.2 | - | - |  |
| HSP40 | YJL073W | P40358 | 447.8 | 3181.9 | 2.5 | - | - | 0.6 | 2.8 | - | - |  |
| HSP40 | YLR090W | P39102 | 327.2 | 1211.7 | 4.5 | 37.1 | 28.2 | 84.5 | 54.9 | - | - |  |
| HSP40 | YNL328C | P42834 | 250.4 | 572.6 | 0.9 | - | 24.8 | - | 3.3 | - | - |  |
| HSP40 | YGL128C | P52868 | - | - | 1.1 | - | - | 1.9 | 1.3 | - | - |  |
| HSP40 | YJL162C | P46997 | - | 183.5 | 2.4 | - | - | - | 0.6 | - | - |  |
| HSP40 | YJR097W | P47138 | - | 2010.0 | - | - | 42.6 | 1.4 | 6.8 | - | - |  |
| HSP40 | YPR061C | Q12350 | - | 414.1 | - | - | - | - | 0.3 | - | - |  |
| HSP40 | YMR161W | P48353 | - | 1835.1 | 38.7 | - | 30.7 | 6.8 | 16.9 | - | - |  |
| HSP40 | YNL077W | P53940 | - | 124.5 | 0.0 | - | - | - | 0.1 | - | - |  |
| HSP40 | YNL064C | P25491 | - | 118525.4 | 1190.0 | 1234.8 | 1777.5 | 1811.4 | 1644.3 | 8304.9 | 15534.9 |  |
|  |  | **CV** | **159.0** | **207.7** | **205.1** | **129.9** | **164.0** | **245.3** | **240.1** | **66.7** | **163.1** | **175.7** |
| CCT | YDR188W | P39079 | 5034.8 | - | 219.6 | - | - | 282.5 | 205.8 | 5546.4 | 14066.8 |  |
| CCT | YDL143W | P39078 | 20009.3 | 5535.0 | 218.0 | - | - | 581.8 | 375.1 | 5746.8 | 11696.6 |  |
| CCT | YDR212W | P12612 | 11544.5 | - | 317.0 | - | - | 170.4 | 165.1 | 13568.2 | 10963.9 |  |
| CCT | YIL142W | P39076 | 7072.0 | - | 494.5 | - | - | 229.5 | 237.4 | 14905.8 | 10320.6 |  |
| CCT | YJR064W | P40413 | 6276.9 | - | 500.0 | - | - | 331.4 | 295.2 | - | 10498.6 |  |
| CCT | YJL008C | P47079 | 5977.6 | - | 481.2 | - | - | 424.7 | 342.9 | 3678.0 | 17300.0 |  |
| CCT | YJL111W | P42943 | 2906.9 | - | 331.5 | - | - | 223.7 | 198.0 | 5010.0 | 15241.7 |  |
| CCT | YJL014W | P39077 | - | - | 349.9 | - | - | 282.5 | 234.7 | 10134.2 | 9642.8 |  |
|  |  | **CV** | **68.4** |  | **32.0** |  |  | **41.8** | **28.8** | **53.7** | **22.1** | **41.1** |
| AAA+ | YLL026W | P31539 | 27435.0 | 32808.2 | 932.9 | 988.4 | 995.8 | 461.6 | 677.0 | 30571.7 | 55800.4 |  |
| AAA+ | YDR258C | P33416 | 10460.0 | 2993.4 | 259.7 | 168.6 | 98.7 | 91.2 | 135.2 | 13397.0 | 21354.6 |  |
| AAA+ | YBR227C | P38323 | 1186.5 | 10868.1 | 37.6 | 59.9 | 30.5 | 3.3 | 25.3 | - | 518.8 |  |
|  |  | **CV** | **102.2** | **99.3** | **113.7** | **125.1** | **143.7** | **131.2** | **125.0** |  | **107.8** | **118.5** |

## Table S4. Chaperone and substrate count correlation statistics.

Correlation coefficients between chaperone abundance and the number of substrates, volume of substrates and substrate flux calculated from different quantitation datasets. Both Pearson linear and Spearman rank correlations are shown for comparison.


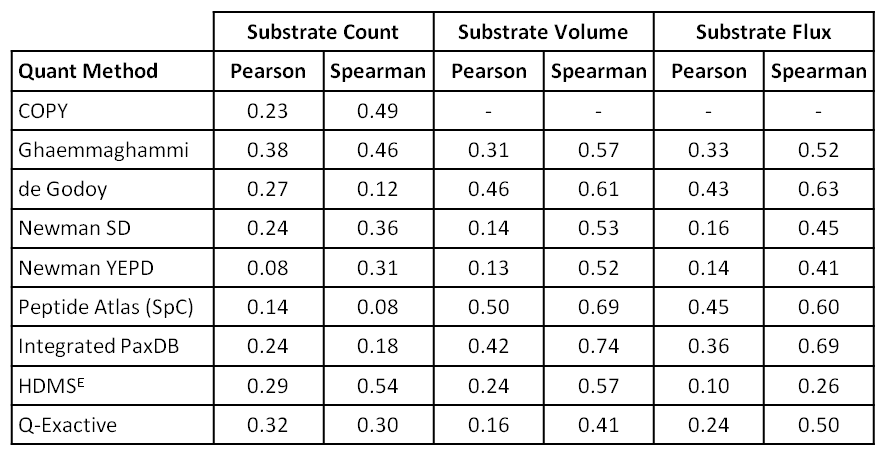


## Table S5. Overall folding for top15 chaperones as determined using Gong and colleagues protein-protein interaction data.

a. Chaperone cpc values were taken from out COPY yeast quantitation; b, as determined for the chaperone integrators from Gong et al. [11]; c, total substrate values were taken from PaxDB for SILAC based quantification determined by de Godoy and colleagues [7] and converted to copies per cell assuming 60 million protein molecules per cell; d, flux was calculated as the product of total substrate volume and degradation rate obtained from the turnover study of Belle at al. [6]

| **Gene Name** | **Systematic ORF Name** | **Chaperone Class** | **Chaperone Abundance (CPC)^a^** | **Number of Substrates^b^** | **Total Substrate Volume (CPC)^c^** | **Total Flux^d^** |
| --- | --- | --- | --- | --- | --- | --- |
| Ssb1 | YDL229W | HSP70 | 68,568.84 | 2512 | 38,948,144.18 | 2,656,394 |
| Ssa1 | YAL005C | HSP70 | 335,459.61 | 1968 | 34,892,319.17 | 2,246,804 |
| Ssa2 | YLL024C | HSP70 | 57,749.94 | 1533 | 24,542,316.64 | 1,800,238 |
| Sse1 | YPL106C | HSP70 | 61,984.14 | 1545 | 27,374,385.23 | 1,763,033 |
| Ssb2 | YNL209W | HSP70 | 85,651.48 | 1001 | 20,648,236.50 | 1,364,517 |
| Hsc82 | YMR186W | HSP90 | 84,372.67 | 369 | 17,011,936.13 | 1,346,202 |
| Hsp82 | YPL240C | HSP90 | 23,038.60 | 687 | 15,087,641.83 | 1,156,089 |
| Ssa4 | YER103W | HSP70 | 5,704.62 | 375 | 14,555,330.12 | 1,041,775 |
| Hsp26 | YBR072W | SMALL | 234,834.03 | 83 | 8,113,500.11 | 849,338 |
| Ssa3 | YBL075C | HSP70 | 658.42 | 262 | 10,222,042.30 | 752,661 |
| Sec63 | YOR254C | HSP40 | 10,766.96 | 113 | 7,844,728.30 | 734,306 |
| Cct8 | YJL008C | CCT | 5,977.62 | 160 | 9,574,088.79 | 698,661 |
| Hsp31 | YDR533C | SMALL | 6,452.20 | 88 | 5,440,859.41 | 697,817 |
| Cct4 | YDL143W | CCT | 20,009.28 | 136 | 8,606,961.46 | 637,563 |
| Cct6 | YDR188W | CCT | 5,034.76 | 85 | 6,835,429.54 | 609,358 |

## Table S6. Overall distribution of chaperone-mediated and independent folding for all yeast proteins.

Data presented both by number, abundance and flux as chaperone targets of un-mediated independent folding proteins**.**

| **Chaperone Class** | **% Proteins^a^** | **%Protein Abundance^b^** | **%Protein Flux^c^** |
| --- | --- | --- | --- |
| **AAA+** | 4.17 | 2.37 | 1.75 |
| **CCT** | 4.00 | 3.22 | 4.14 |
| **HSP40** | 17.15 | 10.09 | 8.34 |
| **HSP60** | 0.79 | 1.11 | 1.60 |
| **HSP70** | 46.98 | 68.12 | 70.61 |
| **HSP90** | 6.44 | 8.60 | 9.02 |
| **PFD** | 15.22 | 3.60 | 2.40 |
| **SMALL** | 5.27 | 2.88 | 2.13 |
| **All** | 36.38 | 57.45 | 61.96 |
| **None** | 63.62 | 42.55 | 38.04 |

^a^Proteins with measured abundance in the most comprehensive quantitation set derived from labelled mass spectrometry (de Godoy et al set).

^b^measured abundance using the de Godoy data set.

^C^calculated using Belle e*t al.* turnover data

## Table S7. Significance testing of total abundance of protein substrate folding mediated by chaperone class.

The modified Fisher Exact test (EASE) was used to estimate p-value significance of the fractional protein volume of protein substrate attributed to individual sub-cellular localisations, with respect to known locations of all proteins. Calculations were scaled by protein abundances taken from the de Godoy set, using assigned locations from Huh et al. Highly significant p-values are highlighted in bold green (p < 0.001), with more modest ones in yellow (p<0.05).

| **Sub-cellular localisation of target proteins** | Chaperone class | | | | | | | |
| --- | --- | --- | --- | --- | --- | --- | --- | --- |
|  | **HSP70** | **AAA+** | **PFD** | **HSP90** | **SMALL** | **CCT** | **HSP40** | **HSP60** |
| actin | 8.2E-01 | 1.0E+00 | 4.4E-02 | 1.0E+00 | 1.0E+00 | 1.0E+00 | 5.2E-01 | 1.0E+00 |
| ambiguous | **7.7E-05** | 4.2E-01 | **5.2E-03** | 4.5E-01 | 3.2E-01 | 1.0E+00 | 6.3E-01 | 1.0E+00 |
| bud | 4.8E-02 | 7.2E-01 | 1.3E-01 | 1.0E+00 | 1.0E+00 | 1.0E+00 | 6.7E-01 | 1.0E+00 |
| bud neck | **1.8E-04** | 3.4E-01 | 9.1E-02 | 5.5E-01 | 5.3E-01 | 1.0E+00 | 7.1E-01 | 1.0E+00 |
| cell periphery | **2.4E-06** | 4.5E-01 | **6.7E-04** | 1.0E+00 | 9.2E-02 | 1.0E+00 | 8.8E-01 | 1.0E+00 |
| cytoplasm | **1.6E-09** | 1.7E-02 | 7.5E-02 | 5.1E-02 | **6.3E-03** | **7.2E-03** | **1.6E-03** | 5.9E-01 |
| endosome | 6.4E-02 | 1.0E+00 | 7.3E-02 | 1.0E+00 | 4.0E-01 | 1.0E+00 | 6.0E-01 | 1.0E+00 |
| ER | 7.6E-01 | 6.5E-02 | **3.9E-03** | 2.5E-02 | 4.8E-02 | 9.0E-02 | **1.3E-15** | 1.0E+00 |
| ER to Golgi | 6.0E-01 | 1.0E+00 | 1.0E+00 | 1.0E+00 | 1.0E+00 | 1.0E+00 | 1.0E+00 | 1.0E+00 |
| Golgi | 8.4E-01 | 1.0E+00 | 1.5E-02 | 4.0E-01 | 1.0E+00 | 1.0E+00 | **2.7E-05** | 1.0E+00 |
| Golgi to ER | 3.8E-01 | 1.0E+00 | 5.8E-01 | 1.0E+00 | 1.0E+00 | 1.0E+00 | **2.1E-07** | 1.0E+00 |
| Golgi to vacuole | 7.0E-01 | 6.4E-01 | 7.6E-01 | 4.0E-01 | 6.4E-01 | 1.0E+00 | 1.0E-01 | 1.0E+00 |
| lipid particle | 1.0E-01 | 1.0E+00 | 1.0E+00 | 1.0E+00 | 1.0E+00 | 1.0E+00 | 1.0E+00 | 1.0E+00 |
| microtubule | 3.7E-02 | 1.0E+00 | **9.7E-04** | 1.0E+00 | 1.0E+00 | 1.0E+00 | 1.0E+00 | 1.0E+00 |
| mitochondrion | **4.0E-06** | 7.8E-01 | **5.1E-07** | **8.5E-03** | 1.0E-01 | 1.9E-01 | **2.1E-03** | **1.0E-03** |
| nuclear periphery | 2.4E-01 | 4.1E-01 | 1.0E-01 | 1.0E+00 | 1.0E+00 | 6.1E-01 | 2.4E-01 | 1.0E+00 |
| nucleolus | **1.2E-06** | 1.0E+00 | 1.3E-01 | 1.0E+00 | 9.3E-02 | 1.2E-02 | 4.7E-01 | 6.3E-01 |
| nucleus | 4.3E-01 | 9.3E-01 | 1.3E-01 | 1.9E-01 | 2.8E-01 | 1.4E-01 | **7.5E-08** | 5.3E-02 |
| peroxisome | 2.2E-02 | 4.4E-01 | 1.0E+00 | 1.0E+00 | 1.2E-01 | 1.0E+00 | 1.0E+00 | 1.0E+00 |
| punctate composite | 3.8E-01 | 1.0E+00 | 1.5E-01 | 1.4E-01 | 6.0E-01 | 4.8E-01 | **1.4E-05** | 2.2E-01 |
| spindle pole | 1.1E-01 | 1.0E+00 | **3.6E-05** | 1.0E+00 | 1.0E+00 | 6.3E-01 | 3.7E-01 | 1.0E+00 |
| vacuolar membrane | **1.4E-04** | 1.0E+00 | 1.8E-01 | 1.0E+00 | 4.2E-01 | 1.0E+00 | 3.4E-01 | 1.0E+00 |
| vacuole | **5.5E-03** | 2.3E-02 | 1.9E-02 | 4.9E-01 | 9.2E-02 | 1.0E+00 | 7.7E-01 | 1.0E+00 |

## Figure S1.A. Light and heavy peptide transition XICs Peptide DVSVEEDISELLR for P40358 .

This protein was observed by COPY,de Godoy and Ghaemmaghammi not by HDMSE, Qexactive and Newman data sets

## Figure S1B. Light and heavy peptide transition XICs for Peptide NTINEASFK for P09435.

This protein was seen by COPY, Ghaemmaghammi, HDMSE and Q-Exactive but not by de Godoy or Newman datasets

**
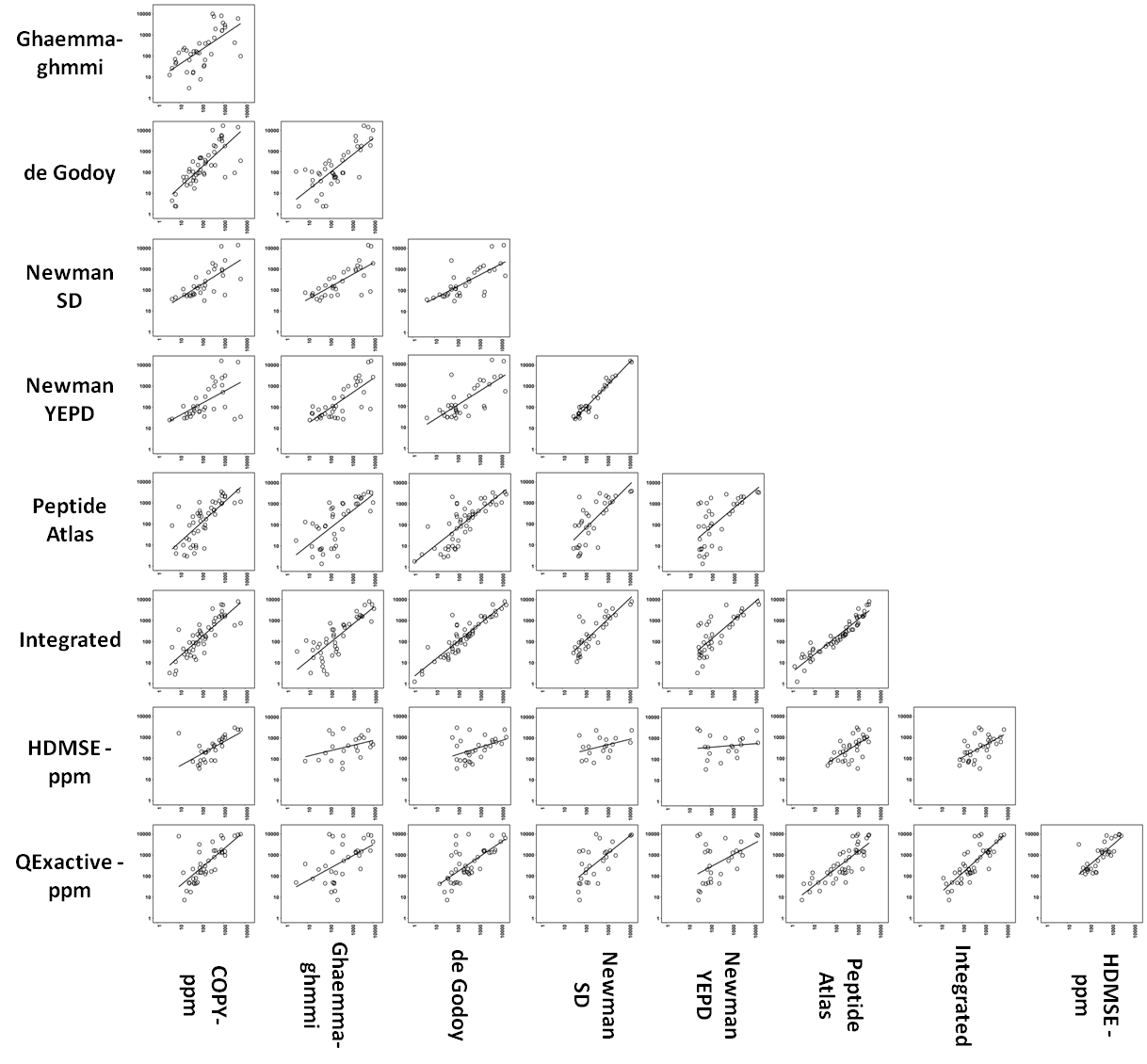
**

## Figure S2. Scatter plot matrix comparing all chaperone abundances across the different quantification methods.


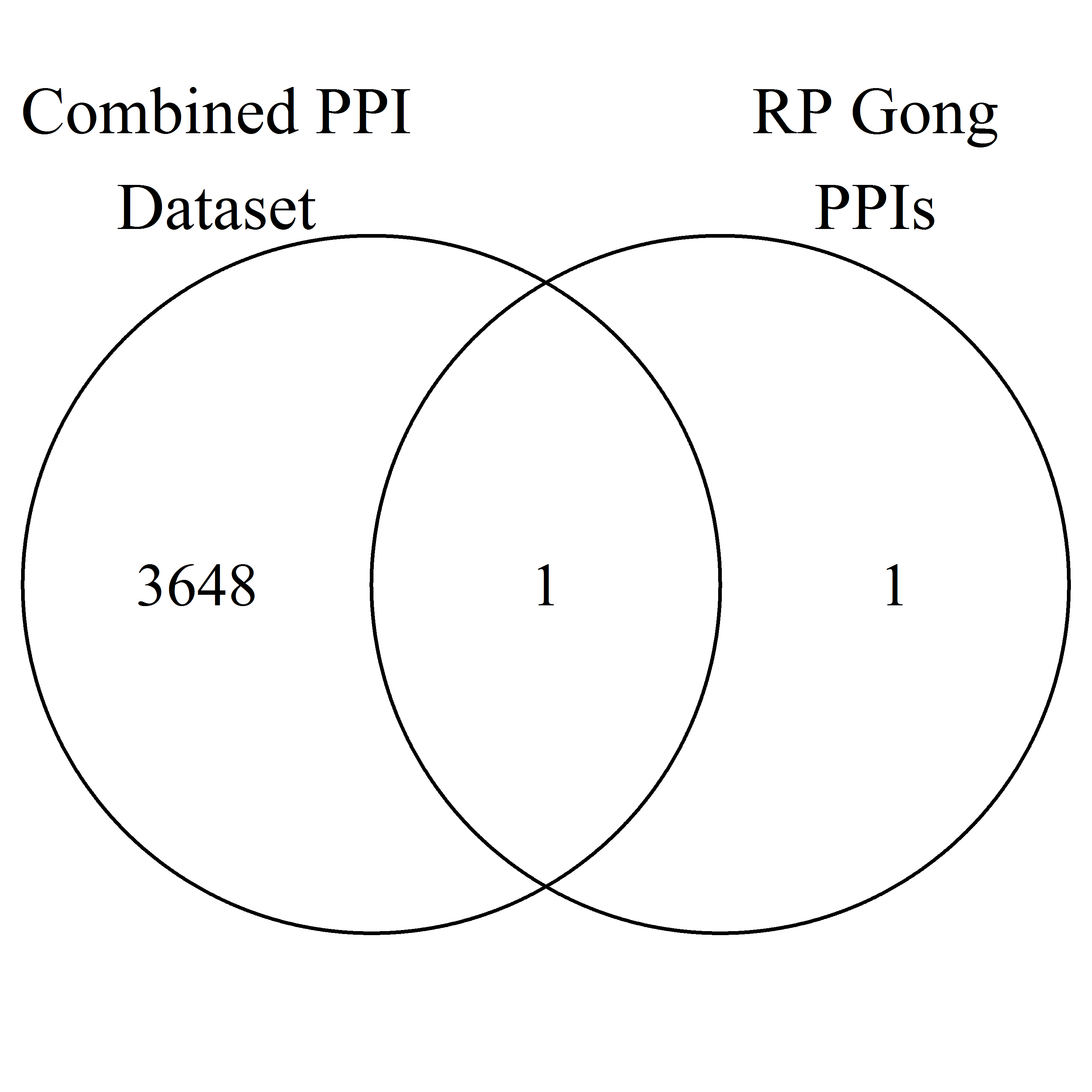

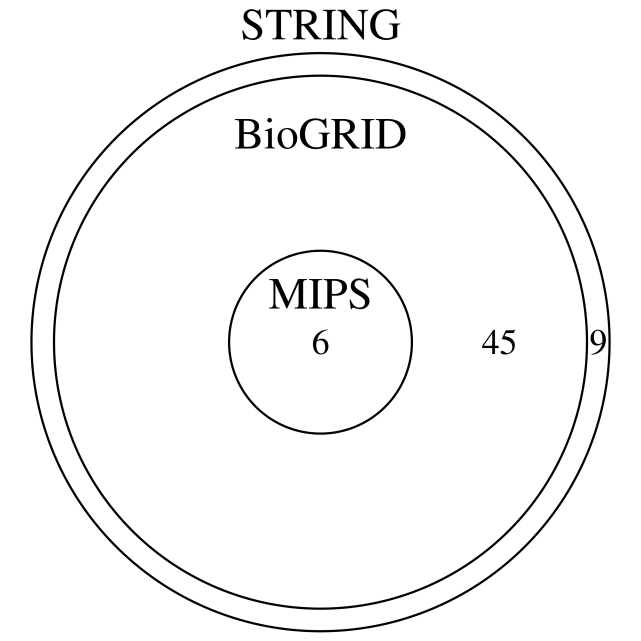

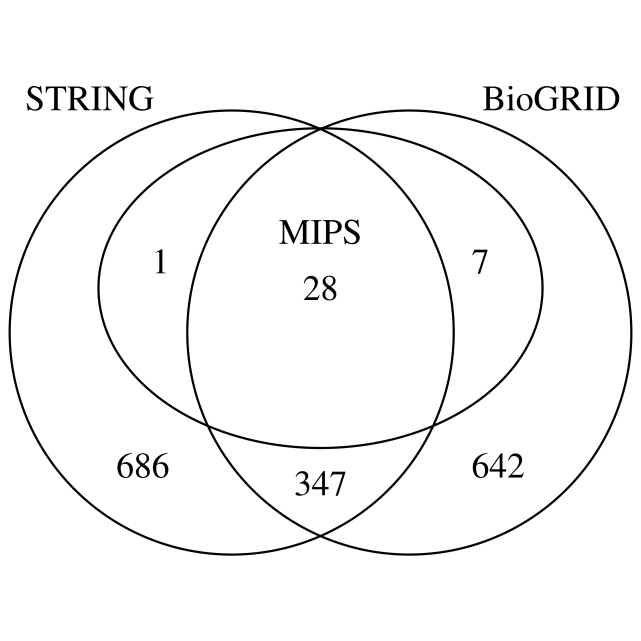

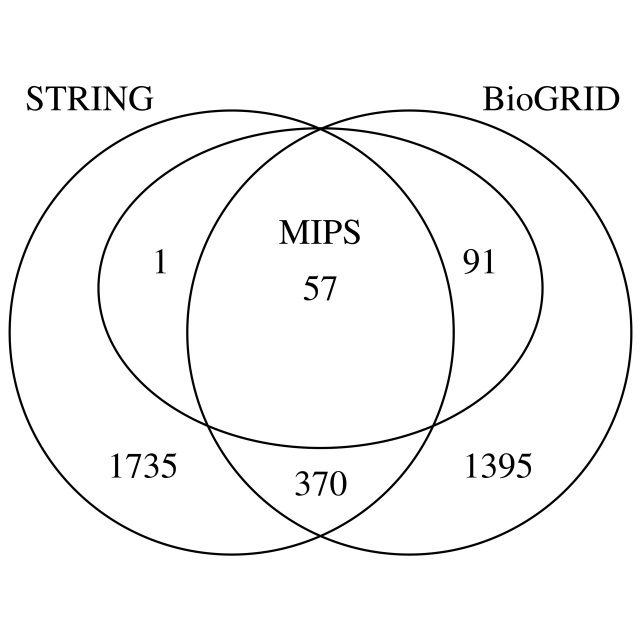


**A**

**C**

**B**

**D**

## Figure S3. Overlap between Protein-Protein interaction datasets used in this study.

Venn Diagrams above show,

1. Chaperone protein overlap between PPI datasets
2. Interacting protein overlap between datasets
3. PPI overlap between datasets
4. PPI overlap between combined datasets and reciprocal interacting Gong PPIs

## Figure S4. Chaperone workload efficiency box-and-whisker plots.

The figures in A and B are identical to Fig6 in the main article, except generated with the PaxDB integrated ppm data (A) and the Q-exactive label-free data (B) and show broadly similar trends. In all cases, the prefoldins appear to be the most efficient class of chaperones, responsible for 5-6X the workload in terms of protein abundance than their own abundance.


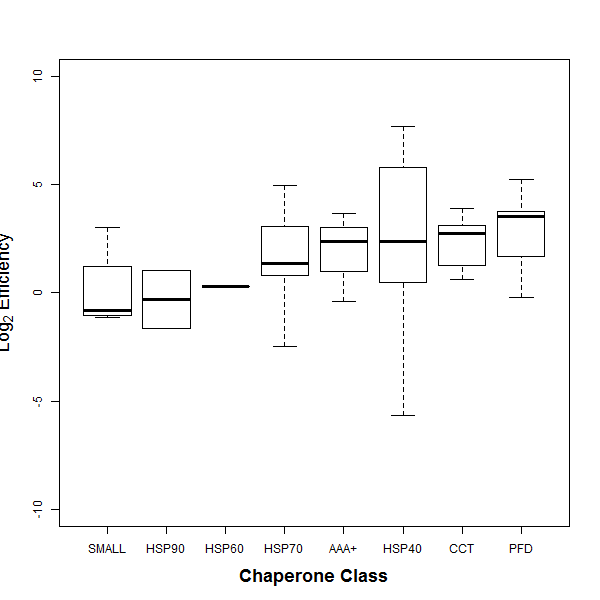


**A.**

**B.
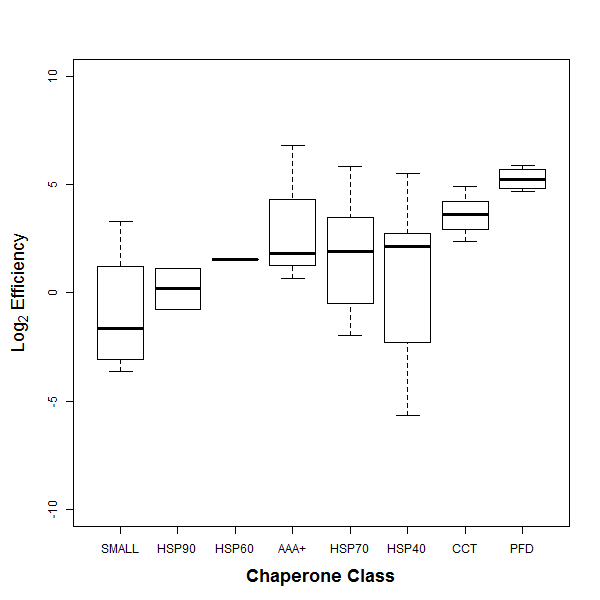
**

## Figure S5. Fraction of chaperone class target abundance classed as essential

Quantitative data taken from the de Godoy study, assigning essentiality to chaperone targets based on the comprehensive yeast deletion project data available via <http://www-sequence.stanford.edu/group/yeast_deletion_project/>. A, essential protein proportions mediated by chaperone classes as determined by total substrate abundance using de Godoy SILAC quant values. B, essential protein proportions mediated by chaperone classes as determined by total substrate flux using turnover data from Belle *et al*. [6]

**
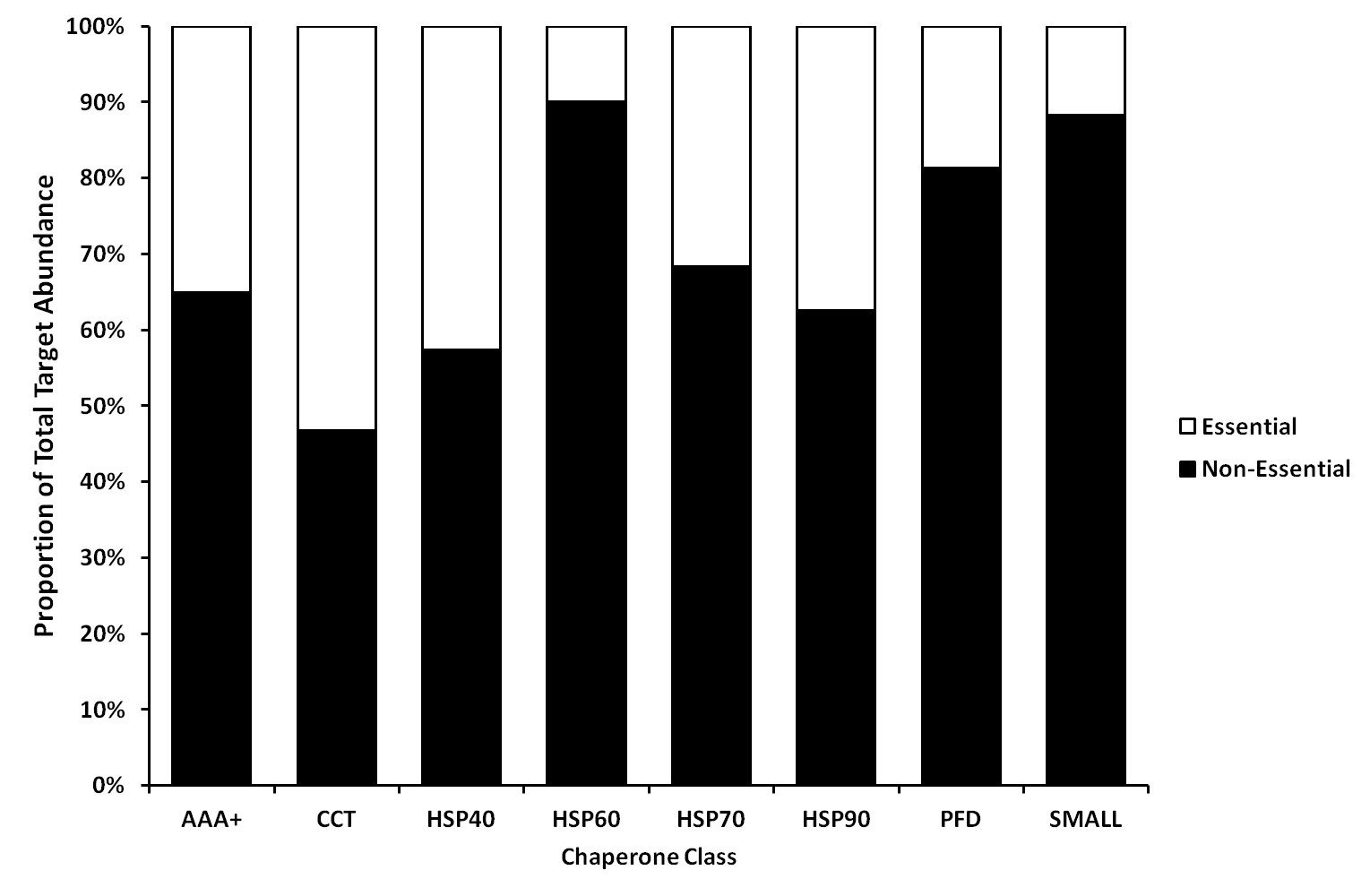
A.**

**B.**

##
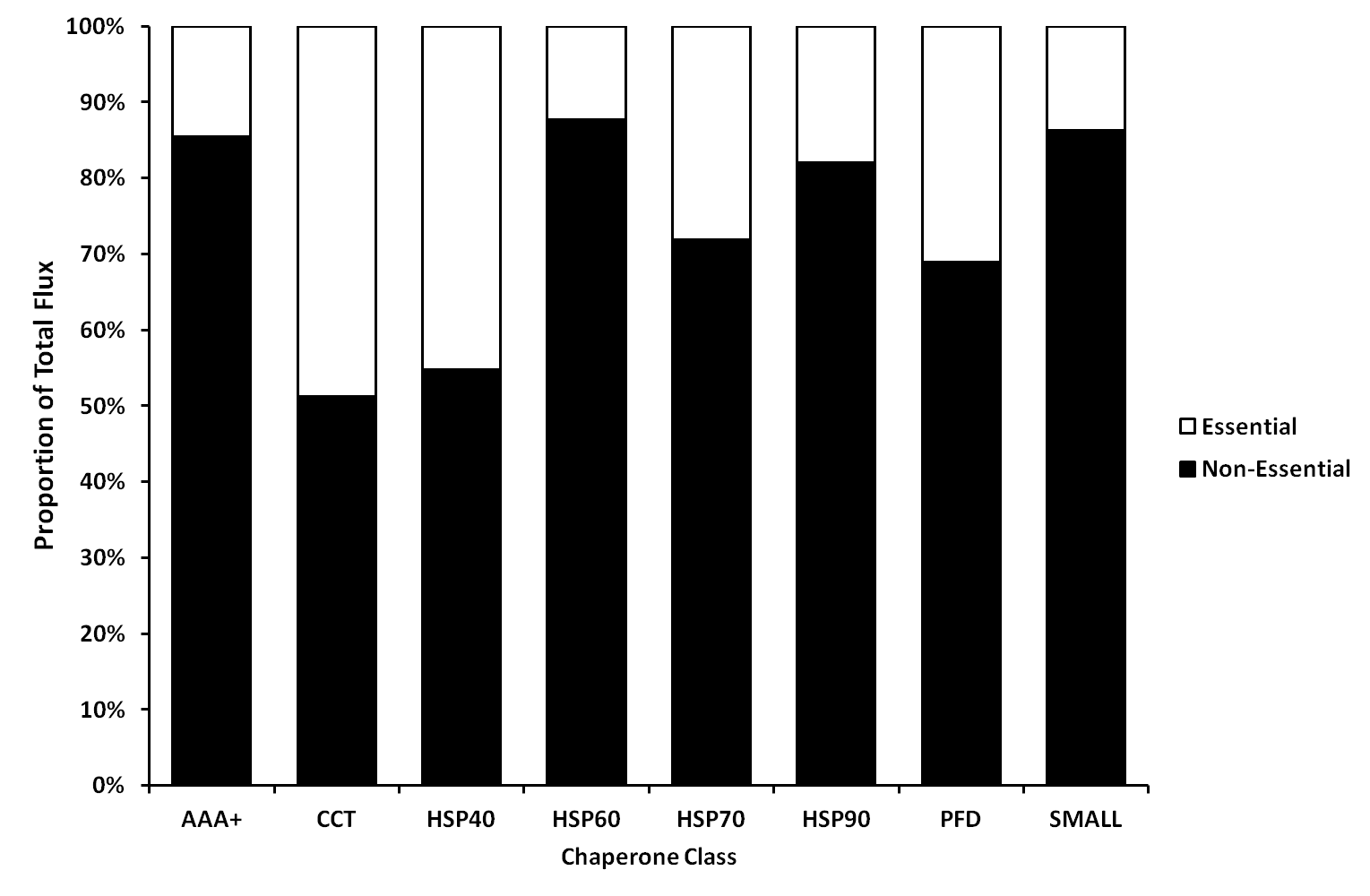


## Figure S6. Proportion of protein abundance of sub-cellular localisation by chaperone mediation and non-mediation .


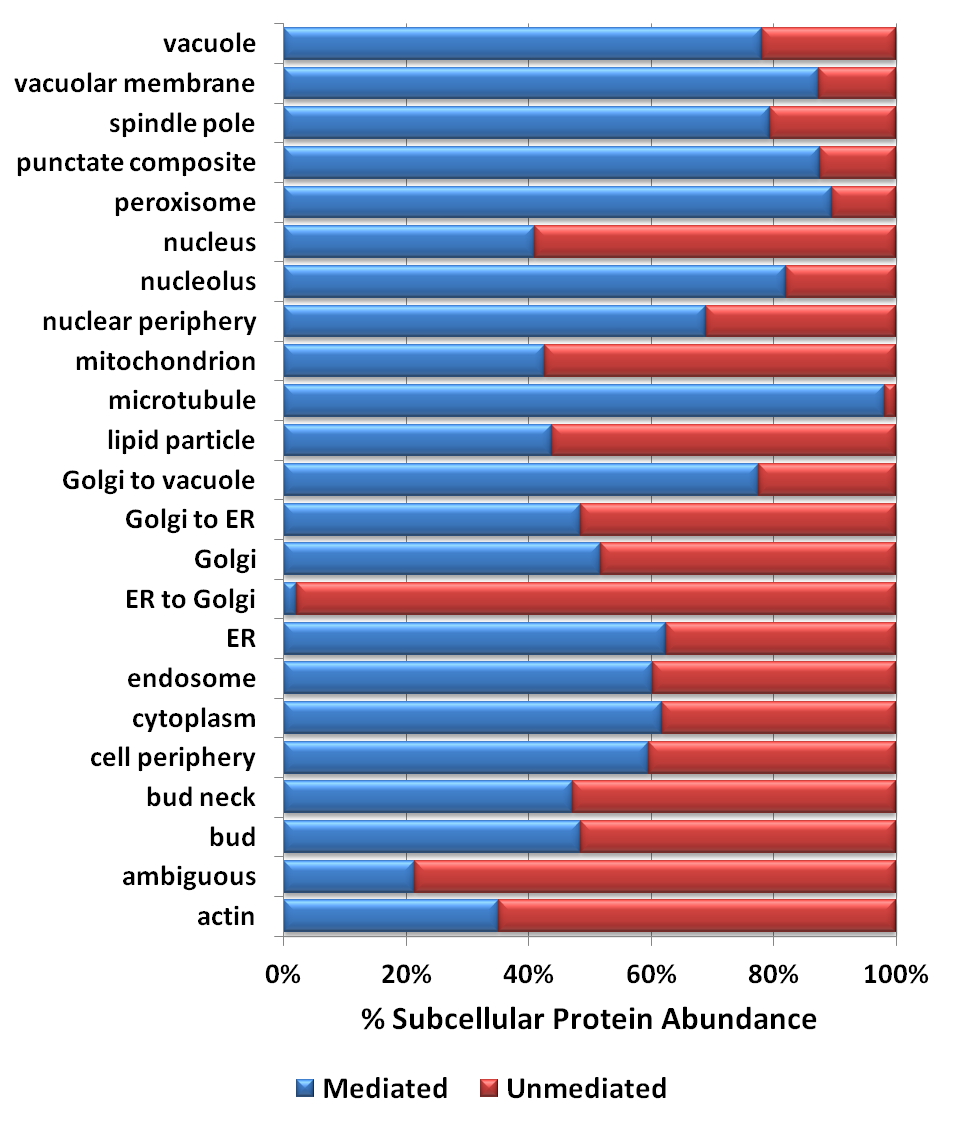


## Figure S7. Proportion of protein abundance of chaperone mediated sub-cellular localisation by chaperone classes.


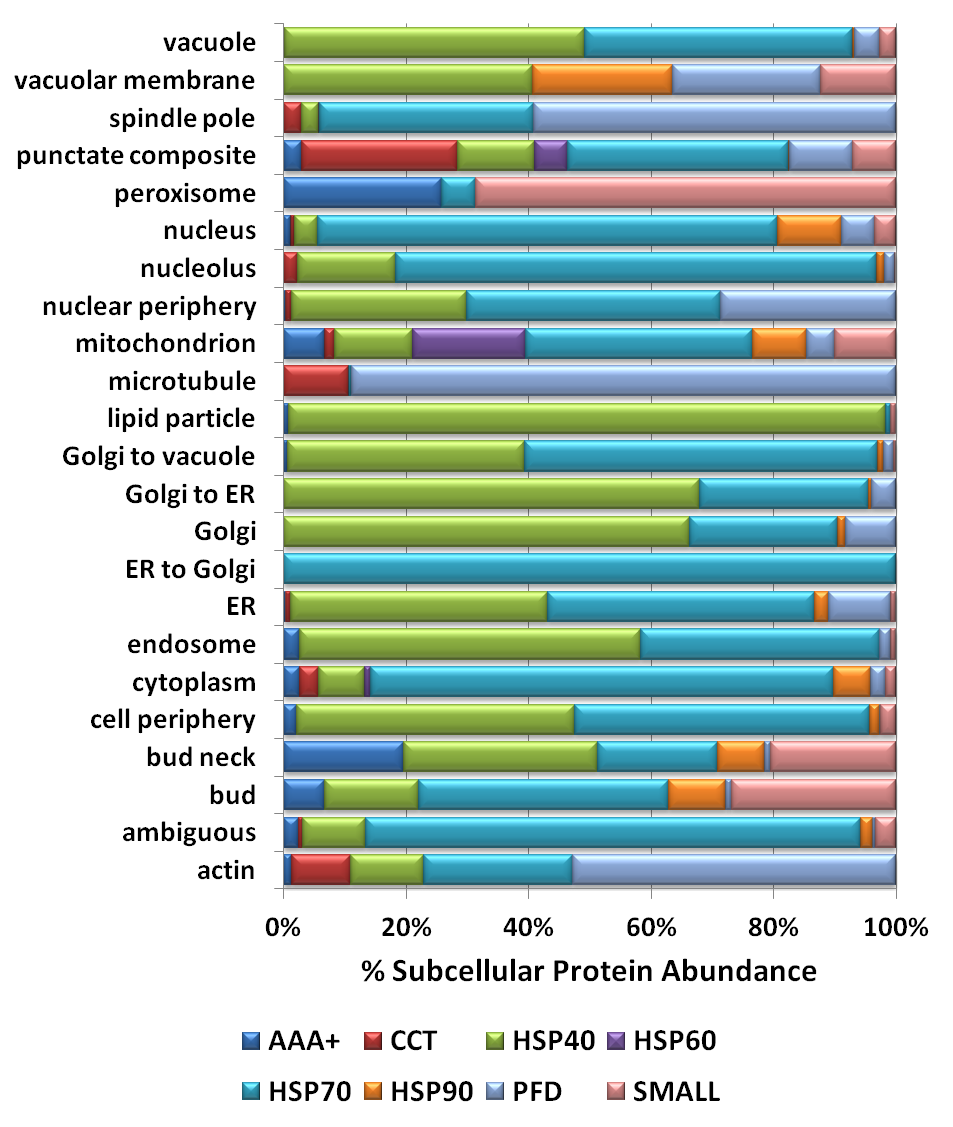

Supplement: Supplementary file 1 — Table S1 Complete set of copies per cell quantitation values obtained for the chaperone proteins Table S2 Spearman Rank correlation matrix between all quantification methods for the chaperone dataset. Table S3 Chaperone quantitation statistics from different studies, including Coefficient of Variation (CV) statistics Table S4 Chaperone and substrate count correlation statistics. Table S5 Overall folding for top15 chaperones as determined using Gong and colleagues protein-protein interaction data. Table S6 Overall distribution of chaperone-mediated and independent folding for all yeast proteins. Table S7 Significance testing of total abundance of protein substrate folding mediated by chaperone class. Figure S1A Light and heavy peptide transition XICs Peptide DVSVEEDISELLR for P40358. Figure S1B Light and heavy peptide transition XICs for Peptide NTINEASFK for P09435. Figure S2 Scatter plot matrix comparing all chaperone abundances across the different quantification methods. Figure S3 Overlap between Protein-Protein interaction datasets used in this study. Figure S4 Chaperone workload efficiency box-and-whisker plots. Figure S5 Fraction of chaperone class target abundance classed as essential Figure S6 Proportion of protein abundance of sub-cellular localisation by chaperone mediation and non-mediation. Figure S7 Proportion of protein abundance of chaperone mediated sub-cellular localisation by chaperone classes. [file pmic0013-1276-SD1.docx]
